# Mechanotransduction activates canonical Wnt/β-catenin signaling to promote lymphatic vascular patterning and the development of lymphatic and lymphovenous valves

**DOI:** 10.1101/gad.282400.116

**Published:** 2016-06-15

**Authors:** Boksik Cha, Xin Geng, Md. Riaj Mahamud, Jianxin Fu, Anish Mukherjee, Yeunhee Kim, Eek-hoon Jho, Tae Hoon Kim, Mark L. Kahn, Lijun Xia, J. Brandon Dixon, Hong Chen, R. Sathish Srinivasan

**Affiliations:** 1 Cardiovascular Biology Research Program, Oklahoma Medical Research Foundation, Oklahoma City, Oklahoma 73104, USA;; 2 Department of Cell Biology, University of Oklahoma Health Sciences Center, Oklahoma City, Oklahoma 73104, USA;; 3 Parker H. Petit Institute for Bioengineering and Bioscience, Georgia Institute of Technology, Atlanta, Georgia 30332, USA;; 4 Department of Biological Sciences, Center for Systems Biology, The University of Texas at Dallas, Richardson, Texas 75080, USA;; 5 Department of Life Science, University of Seoul, Seoul 130-743, Korea;; 6 Department of Medicine, Division of Cardiology, University of Pennsylvania, Philadelphia, Pennsylvania 19104, USA;; 7 Department of Biochemistry, University of Oklahoma Health Sciences Center, Oklahoma City, Oklahoma 73104, USA;; 8 Vascular Biology Program, Boston Children's Hospital, Boston, Massachusetts 02115, USA

**Keywords:** FOXC2, lymphatic valves, lymphatic vascular development, lymphovenous valves, PROX1, Wnt/β-catenin signaling

## Abstract

Lymphatic vasculature regulates fluid homeostasis by returning interstitial fluid to blood circulation. Lymphatic endothelial cells (LECs) are the building blocks of the entire lymphatic vasculature. LECs originate as a homogeneous population of cells predominantly from the embryonic veins and undergo stepwise morphogenesis to become the lymphatic capillaries, collecting vessels or valves. The molecular mechanisms underlying the morphogenesis of the lymphatic vasculature remain to be fully understood. Here we show that canonical Wnt/β-catenin signaling is necessary for lymphatic vascular morphogenesis. Lymphatic vascular-specific ablation of β-catenin in mice prevents the formation of lymphatic and lymphovenous valves. Additionally, lymphatic vessel patterning is defective in these mice, with abnormal recruitment of mural cells. We found that oscillatory shear stress (OSS), which promotes lymphatic vessel maturation, triggers Wnt/β-catenin signaling in LECs. In turn, Wnt/β-catenin signaling controls the expression of several molecules, including the lymphedema-associated transcription factor FOXC2. Importantly, FOXC2 completely rescues the lymphatic vessel patterning defects in mice lacking β-catenin. Thus, our work reveals that mechanical stimulation is a critical regulator of lymphatic vascular development via activation of Wnt/β-catenin signaling and, in turn, FOXC2.

## Abstract (precis)

In this study, Cha et al. show that the Wnt/β-catenin signaling pathway is the link between fluid flow and lymphatic vascular morphogenesis. They provide a molecular and structural framework to study mammalian lymphatic vasculature by demonstrating that mechanical stimulation is a critical regulator of lymphatic vascular development via activation of Wnt/β-catenin signaling.

The lymphatic vasculature collects and returns interstitial fluid to the blood. The mammalian lymphatic vasculature is composed of lymphatic endothelial cells (LECs) that originate predominantly from embryonic veins, although additional sources might exist (Srinivasan et al. 2007; Martinez-Corral et al. 2015; Stanczuk et al. 2015). LEC progenitors from the vein undergo stepwise morphogenesis to form a hierarchical network of capillaries, collecting vessels, intraluminal lymphatic valves (LVs), and lymphovenous valves (LVVs). LVs within collecting vessels regulate the unidirectional flow of lymph, whereas LVVs at the jugular–subclavian vein junctions return lymph to the blood circulation. Defects in lymphatic vessels or valves cause lymphedema, a disease in which tissues swell due to fluid accumulation.

LEC progenitors are specified in a subpopulation of venous ECs by the activation of PROX1 expression. Most of the LEC progenitors migrate out of the vein to form the lymph sacs. However, a subset of LEC progenitors stays on the veins to form four LVVs through the up-regulation of genes in addition to PROX1, such as FOXC2, GATA2, integrin α9 (ITGA9), and ITGA5 (Srinivasan and Oliver 2011; Geng et al. 2016). Next, lymphatic vessels sprout and migrate out from the lymph sacs in a stereotypic manner to form the primitive lymphatic plexus (Coxam et al. 2014; Martinez-Corral et al. 2015). The primitive lymphatic plexus undergoes maturation to form the hierarchical network of vessels. Maturation involves the pruning of branch points, a reduction in vessel diameter, and the acquisition of mural cell coverage to form the collecting lymphatic vessels (Tammela et al. 2007; Norrmen et al. 2009). Finally, LVs develop within the mature collecting lymphatic vessels. At the molecular level, LVs and LVVs are almost identical (Geng et al. 2016).

PROX1 and FOXC2 are important regulators of lymphatic vascular development. LEC progenitors are not specified in the absence of PROX1. Most *Prox1*^+/−^ pups die soon after birth with severe edema. The surviving *Prox1*^+/−^ pups have LVs; however, LVs are absent in those that die soon after birth (Johnson et al. 2008). Likewise, most of the *Prox1*^+/−^ embryos are completely devoid of LVVs (Srinivasan and Oliver 2011; Geng et al. 2016). However, the surviving *Prox1*^+/−^ pups have LVVs (X Geng, unpubl.). Mutations in *FOXC2* are associated with human lymphedema. The lymphatic plexuses of *Foxc2*^−/−^ embryos are mispatterned and abnormally covered with mural cells, do not undergo proper maturation, and lack LVs and LVVs (Petrova et al. 2004; Geng et al. 2016). A variable, strain-dependent valve phenotype is observed in *Foxc2*^+/−^ embryos. Although some *Foxc2*^+/−^ embryos are completely devoid of LVVs, other mutants have two LVVs rather than the four that are normally observed in controls (Geng et al. 2016). A 50% reduction in the number of LVs is observed in *Foxc2*^+/−^ embryos (Kanady et al. 2015). Despite their critical roles, the mechanisms that control the expression of PROX1 and FOXC2, especially in the valves, have not yet been elucidated.

Lymphatic vessels are exposed to reversing fluid flow (more commonly known as oscillatory shear stress [OSS]) (Dixon et al. 2006, 2007; Sweet et al. 2015; Margaris et al. 2016). In vitro, OSS promotes the expression of FOXC2 in LECs (Sabine et al. 2012; Sweet et al. 2015). Furthermore, the mesenteric lymphatic vessels do not undergo proper maturation, and LVs do not form in *Clec2*^−/−^ mice with defective lymph flow (Sweet et al. 2015). Therefore, OSS was proposed to control lymphatic vessel maturation and LV formation. However, how OSS is sensed and translated into FOXC2 expression is not known.

Intriguingly, OSS is not sufficient to promote PROX1 expression in LECs (Sabine et al. 2012; Sweet et al. 2015). Additional signaling pathways are likely required to regulate PROX1 expression in the valves. The Wnt/β-catenin pathway regulates PROX1 expression in colon cancer cells and neural stem cells (Petrova et al. 2008; Karalay et al. 2011). Recently, activation of the Wnt/β-catenin pathway was shown to regulate PROX1 expression in the lymphatic vasculature of zebrafish embryos (Nicenboim et al. 2015). Using cell-based approaches and mouse models, we show that the Wnt/β-catenin pathway regulates PROX1 expression in valves. Additionally, we identified unanticipated roles for this pathway in OSS sensing, lymphatic vascular patterning, and FOXC2 expression.

## Results

### Wnt/β-catenin signaling is active in developing LVVs, LVs, and VVs

**Figure 1.**
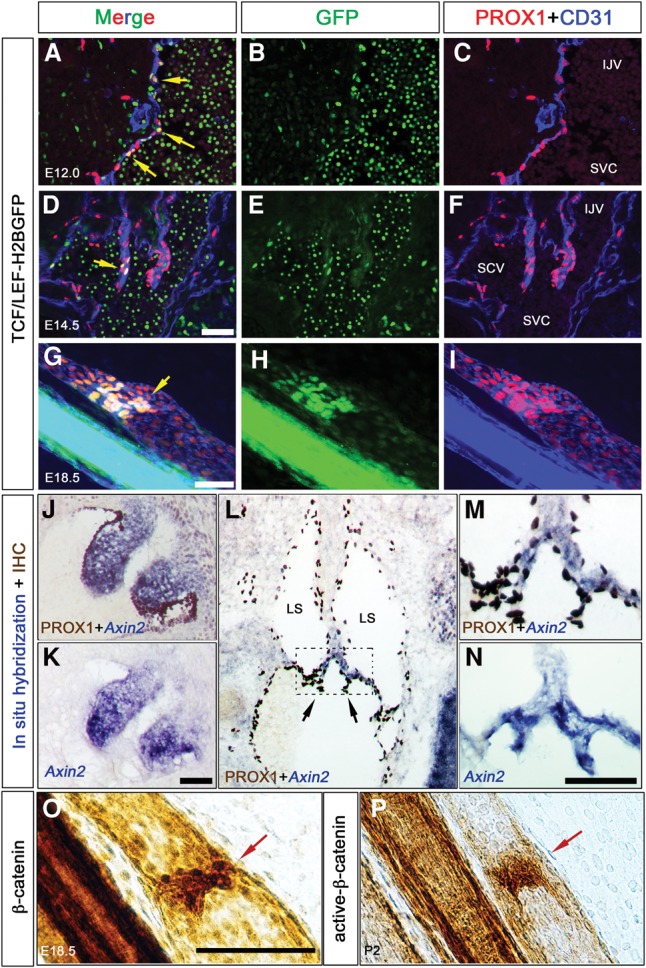
The canonical Wnt/β-catenin signaling pathway is active in the developing LVVs and LVs. (*A*–*I*) TCF/LEF-H2BEGFP embryos were collected at E12.0 (*A*–*C*), E14.5 (*D*–*F*), and E18.5 (*G*–*I*). E12.0 and E14.5 embryos were frontally sectioned, and IHC was performed for GFP, PROX1, and the pan-EC marker CD31. The mesenteries of E18.5 embryos were analyzed by whole-mount IHC for the same markers. The canonical Wnt/β-catenin signaling pathway is active in the GFP^+^ cells of the LVVs (*A*–*F*, yellow arrows) and LVs (*G*–*I*, yellow arrow). (*J*–*N*) The expression of *Axin2*, a target of Wnt/β-catenin signaling pathway, was analyzed in E14.5 embryos by in situ hybridization. Adjacent sections were coimmunostained for PROX1. (*J*,*K*) PROX1^+^ ECs and the mesenchyme of the semilunar valves of the heart are *Axin2*^+^. (*L*–*N*) LVVs (arrows) strongly express *Axin2*. (*O*,*P*) The mesenteric lymphatic vessels were immunostained for total β-catenin (*O*) or nonphosphorylated active β-catenin (*P*). LVs are strongly labeled by both antibodies (red arrows). (LS) Lymph sacs; (IJV) internal jugular vein; (SCV) subclavian vein; (SVC) superior vena cava. Bars: *A–F*,*J–P*, 100 µm; *G*–*I*, 50 µm. *n* = 4 for each experiment.

To determine whether Wnt/β-catenin signaling is active in the mouse lymphatic vasculature, we used transgenic TCF/LEF-H2B-EGFP reporter mice (Ferrer-Vaquer et al. 2010). In these mice, a nuclear H2B-EGFP fusion is under the control of TCF/LEF elements and activated by Wnt/β-catenin signaling. We analyzed 12-µm frontal sections of developing reporter embryos by immunohistochemistry (IHC) and determined that ∼10% of PROX1^+^ LEC progenitors on the vein are H2B-EGFP^+^ at embryonic day 11.0 (E11.0) (Supplemental Fig. 1A). However, LECs outside the vein are only rarely labeled. At E12.0 and E14.5, a subpopulation of LVV-forming ECs (LVV-ECs) express nuclear H2B-EGFP (Fig. 1A–F). At E15.5, VV-forming ECs (VV-ECs) are H2B-EGFP^+^ (Supplemental Fig. 1B). To analyze LVs, we performed whole-mount IHC of the mesentery of E16.5–E18.5 TCF/LEF-H2B-EGFP embryos. We identified modest H2B-EGFP expression in LECs at all stages (Fig. 1G–I; Supplemental Fig 1C,D). However, H2B-EGFP expression is consistently stronger in the LV-forming ECs (LV-ECs) at the three obvious stages of LV formation; i.e., up-regulation of PROX1 expression, formation of the circular sheath, and a mature valve.

EGFP expression in the LVV-ECs is mosaic. Transgenic reporters do not fully recapitulate the activity of the Wnt/β-catenin signaling pathway in vivo (Al Alam et al. 2011). Indeed, it was previously reported that a different Wnt/β-catenin signaling pathway reporter, BAT-gal, is inactive in LVs (Norrmen et al. 2009), and we did not observe any BAT-gal^+^ cells in the entire lymphatic vasculature (data not shown). Therefore, we used additional approaches to assess Wnt/β-catenin signaling activity in the valves. We performed in situ hybridization for *Axin2*, a well-characterized target of the Wnt/β-catenin signaling pathway, in numerous contexts. As a positive control, we evaluated cardiac semilunar valves, which display activated Wnt/β-catenin signaling (Fig. 1J,K; Alfieri et al. 2010). Using this approach, we observed strong and uniform expression of *Axin2* in LVVs at E14.5 (Fig. 1L–N). Furthermore, we analyzed mesenteric lymphatic vessels by IHC with antibodies that recognize total β-catenin or nonphosphorylated (active) β-catenin and clearly detected the LVs (Fig. 1O,P).

Together, these results suggest that the Wnt/β-catenin signaling pathway is active in LECs and particularly in the LVs, LVVs, and VVs, in vivo.

### β-Catenin is required for valve development

To elucidate the importance of the Wnt/β-catenin signaling pathway in valve development, we first conditionally deleted *Ctnnb1* (the gene encoding β-catenin) using *Lyve1-Cre* mice (Brault et al. 2001; Pham et al. 2010). Lineage tracing with an *R26*^+/*tdTomato*^ reporter revealed that *Lyve1-Cre* is active in the cardinal vein as early as E9.5, and LVVs and lymphatic vessels are efficiently labeled in Cre reporter mice at E14.5 (data not shown). LVs and VVs that develop at later time points are also labeled by *Lyve1-Cre*. We bred *Lyve1-Cre;Ctnnb1*^+/*f*^ mice with *Ctnnb1*^+/*f*^ mice and failed to obtain any surviving *Lyve1-Cre;Ctnnb1*^*f/f*^ (*Lyve1-Cre;Ctnnb1*^*LOF*^) pups from >200 pups analyzed at postnatal day 0 (P0). Instead, we observed several dead, cyanotic, and edematous *Lyve1-Cre;Ctnnb1*^*LOF*^ pups in the cages, suggesting perinatal lethality. We collected E14.5, E16.5, and E18.5 *Lyve1-Cre;Ctnnb1*^*LOF*^ embryos and determined that they had severe edema (Supplemental Fig. 2A,B; data not shown). Occasionally, some blood was observed in the peripheral skin of the mutant embryos (Supplemental Fig. 2C). We found that the lymph sacs of these embryos were severely dilated, resulting in the constriction of the surrounding veins (Supplemental Fig. 2D–F).

**Figure 2.**
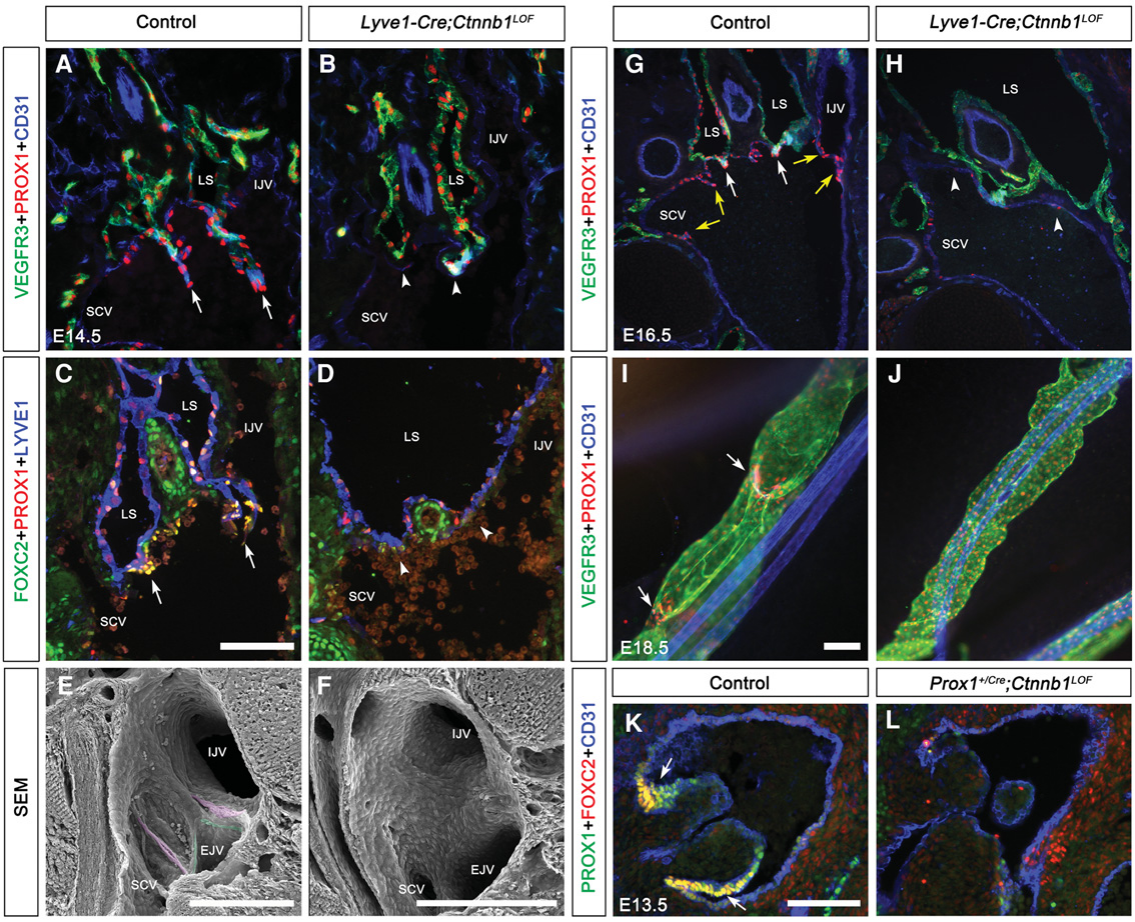
β-Catenin is necessary for the development of LVVs, LVs, VVs, and cardiac valves. (*A*–*J*) The gene encoding β-catenin (*Ctnnb1*) was deleted from LECs, LVV-ECs, LV-ECs, and VV-ECs using *Lyve1-Cre* mice*.* IHC was performed for the indicated markers. Frontal sections from E14.5 (*A*–*D*) and E16.5 (*G*,*H*) embryos revealed the LVVs in controls (arrows) but not in their *Lyve1-Cre; Ctnnb1*^*LOF*^ littermates. Arrowheads point to the valve-forming area of mutants. (*G*,*H*) At E16.5, VVs are seen in control embryos (yellow arrows) but not in mutants. (*E*,*F*) SEM confirmed the presence of LVV-ECs (magenta) and VV-ECs (green) in E14.5 control embryos and their absence in *Lyve1-Cre;Ctnnb1*^*LOF*^ littermates. (*I*,*J*) Whole-mount IHC of the mesenteric lymphatic vessels revealed the presence of LVs in E18.5 control (arrows) but not *Lyve1-Cre;Ctnnb1*^*LOF*^ littermates. (*I*) Furthermore, PROX1 is strongly expressed in the LVs but down-regulated elsewhere in the lymphatic vessels of controls. (*J*) In contrast, PROX1 expression is uniformly high in the lymphatic vessels of the mutants. (*K*,*L*) *Prox1*^+/*Cre*^ was used to delete *Ctnnb1* from the PROX1^+^ cells of the cardiac semilunar valves. IHC was performed for the indicated markers. E13.5 *Prox1*^+/*Cre*^;*Ctnnb1*^*LOF*^ embryos lacked the PROX1^+^FOXC2^+^ cells of the cardiac valves (arrows). (LS) Lymph sacs; (IJV) internal jugular vein; (SCV) subclavian vein; (SVC) superior vena cava. Bars: *A*–*J*, 200 µm; *K*,*L*, 100 µm. *n* = 4 for each experiment.

We recently described the stepwise morphogenesis of LVVs and reported that the PROX1^high^ FOXC2^high^ GATA2^high^ LVV-ECs are first observed at E12.0 (Geng et al. 2016). We found that LVV-ECs are absent in E12.0 *Lyve1-Cre;Ctnnb1*^*LOF*^ embryos (Supplemental Fig. 3A–F). In scanning electron microscopy (SEM) images of control embryos, LVV-ECs could be seen delaminating from the walls of the vein and loosely aggregating with each other; however, these cells are absent in *Lyve1-Cre;Ctnnb1*^*LOF*^ embryos (Supplemental Fig. 3A–F). These results demonstrate that β-catenin is necessary for the differentiation of LVV-ECs. Consistently, IHC revealed that PROX1^high^ FOXC2^high^ GATA2^high^ LVV-ECs are present in E14.5 control embryos but absent in their *Lyve1-Cre;Ctnnb1*^*LOF*^ littermates (Fig. 2A–D; data not shown). SEM confirmed that while LVVs are present in E14.5 control embryos (Fig. 2E, magenta), they are absent in embryos lacking β-catenin (Fig. 2F). Analysis of E16.5 control and *Lyve1-Cre;Ctnnb1*^*LOF*^ embryos revealed that LVV-ECs are absent in mutant embryos at this stage as well (Fig. 2G,H). Thus, *Lyve1-Cre;Ctnnb1*^*LOF*^ embryos display a complete lack, and not just a delay, of LVV-EC differentiation.

At E14.5, VV-ECs start to differentiate and could be seen delaminating from the rim of the venous junction in control embryos (Fig. 2E, green; Geng et al. 2016). These cells rapidly develop to form the VVs at E16.5 (Fig. 2G, yellow arrows). However, VV-ECs are absent in *Lyve1-Cre;Ctnnb1*^*LOF*^ embryos at both the E14.5 and E16.5 stages (Fig. 2F,H, respectively), demonstrating that β-catenin is also necessary for the differentiation of VV-ECs.

LV formation occurs in a stepwise manner starting at E16.5 with the up-regulation of PROX1, FOXC2, and GATA2 in a subset of cells within the lymphatic vessels (Bazigou et al. 2009; Norrmen et al. 2009; Kazenwadel et al. 2015; Sweet et al. 2015). We determined that the PROX1^high^ FOXC2^high^ GATA2^high^ LV-ECs are absent in the mesenteric lymphatic vessels of E16.5 *Lyve1-Cre;Ctnnb1*^*LOF*^ embryos, indicating that β-catenin is also necessary for the differentiation of LV-ECs (Supplemental Fig. 4C–E). At E17.5, LV rudiments are more clearly visible in control embryos and are not observed in mutants (Supplemental Fig. 4A,B,F,G). By E18.5, oval-shaped lymphangions are clearly distinguishable in control mesenteries, and LVs with PROX1^high^ cells are seen. In contrast, the lymphatic vessels of E18.5 *Lyve1-Cre;Ctnnb1*^*LOF*^ embryos are dilated, and LV-ECs are only rarely observed (Fig. 2I,J; data not shown). Consistently, LVs are significantly reduced in the mesenteries of E18.5 *Lyve1-Cre;Ctnnb1*^*LOF*^ embryos (Supplemental Fig. 4C).

We previously reported the presence of PROX1^+^ FOXC2^+^ cells on the downstream side of cardiac valves (Srinivasan and Oliver 2011). These cells also express additional LV, LVV, and VV markers such as ITGA9 and GATA2. We wanted to determine whether the Wnt/β-catenin signaling pathway is necessary for the development of these cells as well. Cre is not expressed in the cardiac valve cells of *Lyve1-Cre* mice (data not shown); therefore, we used previously reported *Prox1*^+/*Cre*^ mice to conditionally delete *Ctnnb1* in these cells. *Prox1*^+/*Cre*^ mice are heterozygous for *Prox1* and lack LVVs, LVs, and VVs (Srinivasan and Oliver 2011). However, PROX1^+^ FOXC2^+^ cells are observed in the cardiac valves of *Prox1*^+/*Cre*^ embryos, and lineage tracing experiments revealed that Cre is active in the PROX1^+^ cells of the cardiac valves but not the cardiac mesenchyme (data not shown). Hence, *Prox1*^+/*Cre*^ will specifically delete *Ctnnb1* in the PROX1^+^ ECs of the cardiac valves. Importantly, the well-characterized roles of β-catenin during cardiac cushion formation are unperturbed (Liebner et al. 2004). We bred *Prox1*^+/*Cre*^;*Ctnnb1*^+/*f*^ and *Ctnnb1*^+/*f*^ mice to obtain *Prox1*^+/*Cre*^;*Ctnnb1*^*LOF*^ embryos and determined that they do not survive past E13.5. Analysis of the cardiac valves in these mutants revealed that the PROX1^+^ FOXC2^+^ cells are absent in the cardiac valves, although the cardiac cushion appears unremarkable (Fig. 2K,L).

Combined, these results conclusively show that β-catenin is necessary for the differentiation of all types of PROX1^high^ FOXC2^high^ GATA2^high^ valvular ECs (VECs).

### β-Catenin regulates the patterning of the lymphatic vasculature

PROX1 and VEGFR3 are expressed in a heterogeneous manner in the mesenteric lymphatic vessels of E18.5 control embryos. They are strongly expressed in the LVs, whereas their expression is much lower in the rest of the lymphatic vessels (Fig. 2I). In contrast, the valveless lymphatic vessels of *Lyve1-Cre;Ctnnb1*^*LOF*^ littermates express uniformly high levels of PROX1 and VEGFR3 (Fig. 2J). This is characteristic of lymphatic vessels that have failed to undergo proper maturation (Norrmen et al. 2009). We wanted to determine whether this phenotype is a consequence of defects in the earlier stages of lymphatic vascular development. We addressed this question using the primitive lymphatic plexus of the peripheral skin as a model.

**Figure 3.**
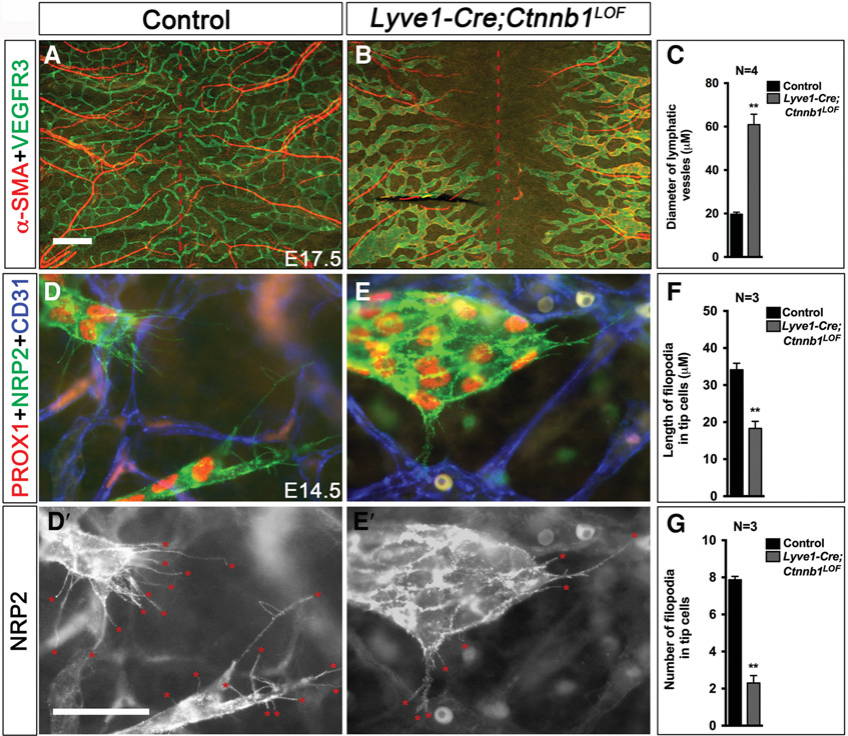
β-Catenin is necessary for the patterning of lymphatic vessels. (*A*–*G*) E17.5 and E14.5 *Lyve1-Cre;Ctnnb1*^*LOF*^ and its control littermates were harvested, and the lymphatic vessels of dorsal skin were analyzed by whole-mount IHC for the indicated markers. (*A*,*B*) The lymphatic vessels of control embryos have reached and crossed over the midline (red dotted line) to form a network of vessels. In contrast, the lymphatic vessels of the mutants are dilated and have not reached the midline. Additionally, abnormal recruitment of α-SMA^+^ mural cells is visible in the lymphatic vessels of mutants. The lymphatic vessel diameter is quantified in *C*. (*D*,*E*) The lymphatic vessels at the leading edge are thin and elongated in E14.5 control embryos. In contrast, they are dilated in *Lyve1-Cre;Ctnnb1*^*LOF*^ embryos. *D*′ and *E*′ show NRP2 expression alone from the corresponding pictures in *D* and *E*, respectively. NRP2 clearly labels the filopodia (red asterisks) on the tip cells of the growing lymphatic vessels. The length (*F*) and the number (*G*) of filapodia are significantly reduced in the mutant embryos. Bars: *A*,*B*, 500 µm; *D*–*E*′, 50 µm. *n* = 4 for each experiment. (**) *P* < 0.01.

In the peripheral skin, the lymphatic vessels start migrating in both the dorsal and ventral directions, approximately from the region where the limbs are located (Coxam et al. 2014; Martinez-Corral et al. 2015). In E17.5 control embryos, the lymphatic vessels have crossed the midline in the dorsal skin and formed a uniform network (Fig. 3A). This lymphatic capillary network is devoid of α-smooth muscle actin^+^ (α-SMA^+^) cell coverage. In contrast, the lymphatic vessels of *Lyve1-Cre;Ctnnb1*^*LOF*^ littermates are dilated well-short of the midline and are abnormally covered with α-SMA^+^ mural cells (Fig. 3B,C).

In order to study the progression of the vascular patterning phenotype, we analyzed the peripheral skin of E14.5 *Lyve1-Cre;Ctnnb1*^*LOF*^ embryos and their control littermates. The lymphatic vessels are rapidly growing toward the midline at this stage and provide an opportunity to understand the mechanisms of lymphatic vascular patterning. As in E17.5 embryos, the lymphatic vessels of E14.5 *Lyve1-Cre;Ctnnb1*^*LOF*^ littermates were further away from the dorsal midline compared with controls (data not shown). Tip cells located at the forefront of vessels dictate vascular patterning with their numerous filopodia, which sense the environment and determine growth and branching. We found that the number of filopodia per tip cell and the length of filopodia are significantly reduced in *Lyve1-Cre;Ctnnb1*^*LOF*^ embryos (Fig. 3D–G).

We also analyzed the lymphatic vessels of the heart. Lymphatic vessels start migrating from the atrial end of the heart at around E14.5 (data not shown). In E17.5 control embryos, lymphatic vessels are seen running from the atrium until the ventricular tip of the hearts (Supplemental Fig. 5A, inset). In contrast, the lymphatic vessels of *Lyve1-Cre;Ctnnb1*^*LOF*^ littermates are dilated with bulbous architecture (Supplemental Fig. 5B). These abnormal lymphatic vessels have migrated to only the middle of the heart (Supplemental Fig. 5B, inset).

These results show that Wnt/β-catenin signaling is necessary for the proper patterning of lymphatic vessels. Specifically, the lymphatic vessels of *Lyve1-Cre;Ctnnb1*^*LOF*^ embryos have a migration defect and are dilated with an abnormal bulbous architecture.

### Wnt/β-catenin signaling regulates the expression of multiple targets in the lymphatic vessels

**Figure 4.**
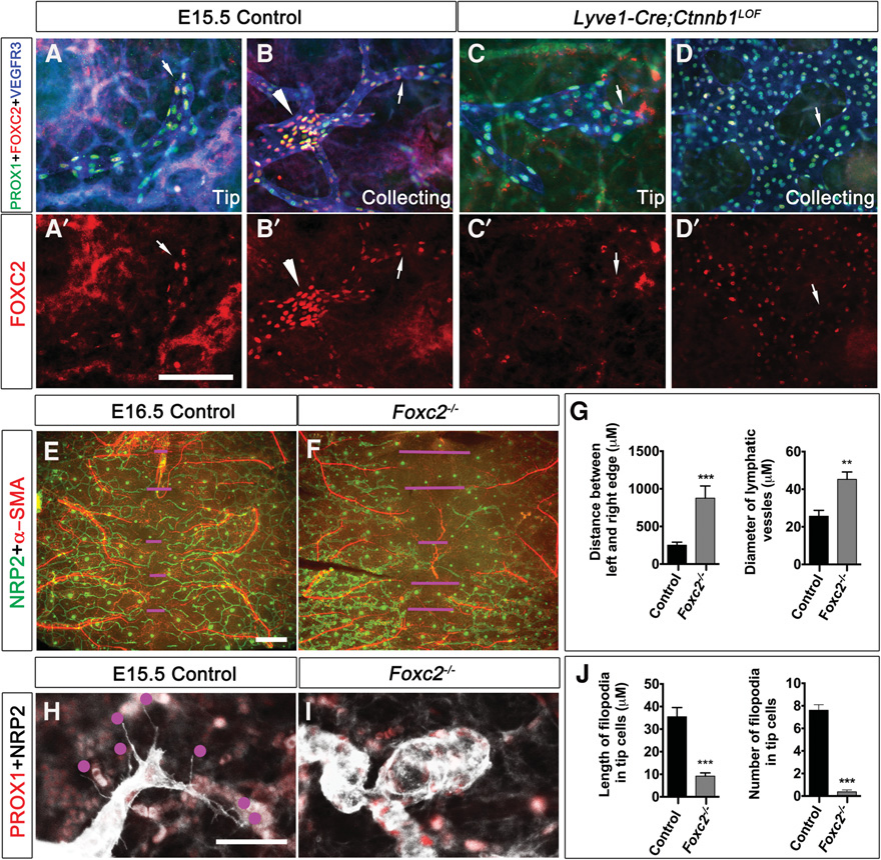
The Wnt/β-catenin signaling pathway regulates FOXC2 expression in LECs. (*A*–*D*) The dorsal skin of E15.5 control (*A*,*B*) and *Lyve1-Cre;Ctnnb1*^*LOF*^ (*C*,*D*) embryos was analyzed by whole-mount IHC for the indicated markers. FOXC2 is expressed in both the tip (*A*,*A*′, arrows) and collecting lymphatic vessels (*B*,*B*′, arrows) of control embryos. (*B*,*B*′) Rudimentary LVs are enriched for FOXC2 (arrowhead). (*C*,*C*′) FOXC2 expression is dramatically down-regulated in the tip cells (arrows) of mutants. (*D*,*D*′) A modest down-regulation of FOXC2 expression is observed in the collecting lymphatic vessels of mice lacking β-catenin (arrows). Furthermore, LV rudiments are absent in *Lyve1-Cre;Ctnnb1*^*LOF*^ embryos. (*E*–*G*) The lymphatic vessels of the skin from E16.5 control and *Foxc2*^−/−^ littermates were analyzed by IHC using the indicated markers. The magenta lines indicate the distance between the tip cells and the opposing front. (*G*) This distance is significantly increased in *Foxc2*^−/−^ embryos, indicating lymphatic vascular hypoplasia. The diameter of lymphatic vessels is also significantly increased in *Foxc2*^−/−^ embryos. (*H*–*J*) The tip cells of E15.5 control embryos have numerous well-formed filopodia (red dots). In contrast, the tip cells of *Foxc2*^−/−^ embryos have a bulbous architecture and hardly any filopodia. The number and length of filopodia in control and *Foxc2*^−/−^ embryos are quantified in *J*. Bars: *A*–*D*, 100 µm; *E*,*F*, 500 µm; *H*,*I*, 25 µm. *n* = 4 for each experiment. (**) *P* < 0.01; (***) *P* < 0.001.

We analyzed control and *Lyve1-Cre;Ctnnb1*^*LOF*^ embryos to identify the Wnt/β-catenin signaling-dependent changes in the lymphatic vessels. Endomucin is a marker that is expressed in venous ECs but not LECs (Fu et al. 2008). Endomucin is not abnormally expressed in the lymph sacs or lymphatic vessels of *Lyve1-Cre;Ctnnb1*^*LOF*^ embryos, suggesting that β-catenin is not necessary for the identity of LECs (Supplemental Fig. 6). In control embryos, PROX1 and FOXC2 are expressed within the lymphatic vasculature in a heterogeneous manner. A modest expression of these molecules is observed in most of the lymphatic vasculature, including the tip cells (Fig. 4A,B, arrows). In contrast, LVs are starting to form at this stage, and expression of PROX1 and FOXC2 is enriched in LV-ECs (Fig. 4B, arrowhead). As expected, LV-ECs are absent in *Lyve1-Cre;Ctnnb1*^*LOF*^ embryos (Fig. 4D). By a semiquantitative measurement of the fluorescent signals, we determined that PROX1 expression is modestly yet significantly down-regulated in the tip LECs (Supplemental Fig. 7). In contrast, PROX1 is up-regulated in the immature collecting lymphatic vessels. A modest down-regulation of FOXC2 expression is observed in the immature collecting lymphatic vessels of *Lyve1-Cre;Ctnnb1*^*LOF*^ embryos (Fig. 4D, arrow). A more dramatic down-regulation of FOXC2 expression is observed in the tip cells of mutants (Fig. 4C, arrow).

Gap junction molecule Connexin37 (CX37) is important for valve formation and also plays an incompletely understood role in lymphatic vascular morphogenesis (Kanady et al. 2011, 2015; Geng et al. 2016). CX37 is expressed in the entire lymphatic vasculature of control embryos. However, CX37 expression is severely down-regulated in the lymphatic vessels of mice lacking β-catenin (Supplemental Fig. 8). These results demonstrate that Wnt/β-catenin signaling regulates the expression of FOXC2, PROX1, and CX37 in LECs.

The phenotypes of *Lyve1-Cre;Ctnnb1*^*LOF*^ embryos are most similar to *Foxc2*^−/−^ embryos. Specifically, both β-catenin and FOXC2 are required for the formation of LVs, LVVs, and VVs and proper lymphatic vessel maturation. To further delineate the functional relationship between β-catenin and FOXC2, we analyzed the lymphatic vascular plexus of *Foxc2*^−/−^ embryos. We found that the lymphatic vessels in the peripheral skin of *Foxc2*^−/−^ embryos do not reach the dorsal midline and are dilated (Fig. 4E–G). Additionally, analysis of the tip cells in *Foxc2*^−/−^ embryos revealed a bulbous architecture with fewer and shorter filopodia in the tip cells, reminiscent of *Lyve1-Cre;Ctnnb1*^*LOF*^ embryos (Fig. 4H–J). Together these results suggested that FOXC2 might be an important target of Wnt/β-catenin signaling in the entire lymphatic vasculature.

### OSS is necessary for the patterning of the lymphatic vasculature

As mentioned above, LECs within the vessel lumen in vivo and in isolated vessel preparations are exposed to OSS (Dixon et al. 2006, 2007; Sweet et al. 2015; Margaris et al. 2016). Approaches to prevent lymph flow and determine its role in lymphatic vascular development currently do not exist. However, the lymphatic vessels of *Clec2*^−/−^ mice are devoid of lymph flow (Sweet et al. 2015). LVVs and VVs that are unaffected by lymph flow form normally in *Clec2*^−/−^ mice (Hess et al. 2014). However, the mesenteric lymphatic vessels of *Clec2*^−/−^ mice do not undergo proper maturation, and LVs do not form (Sweet et al. 2015). These data suggest that OSS is a critical factor involved in lymphatic vascular maturation and LV formation.

Whether the primitive lymphatic plexus of *Clec2*^−/−^ embryos is normal is not known. To address this question, we analyzed the lymphatic vessels in the peripheral skin of *Clec2*^−/−^ embryos and determined that they do not migrate properly and that the tip cells have a bulbous architecture (Supplemental Fig. 9A,B). These phenotypes are strikingly similar to *Foxc2*^−/−^ and *Lyve1-Cre;Ctnnb1*^*LOF*^ embryos. Consistent with this observation, FOXC2 expression is down-regulated in the LECs of *Clec2*^−/−^ embryos (Sweet et al. 2015). We propose that lymphatic vessel migration is an aspect of lymphatic vascular maturation that is regulated by OSS, Wnt/β-catenin signaling, and FOXC2.

### Wnt/β-catenin signaling is necessary and sufficient for OSS-mediated reprogramming of LECs

An in vitro approach has been developed to generate valve-like cells based on the fact that LVs frequently form at sites exposed to OSS (Sabine et al. 2012). OSS is hypothesized to be important for reprogramming LECs into an LV-EC-like identity (Sabine et al. 2012). Accordingly, primary human LECs cultured under OSS in flow chambers up-regulate the expression of LV-EC markers, including FOXC2 and GATA2 (Sabine et al. 2012; Kazenwadel et al. 2015; Sweet et al. 2015). Hence, OSS-exposed LECs are currently the best models available to study valve development in vitro. We term these cells iVECs (OSS-induced VECs) for simplicity. As mentioned in the previous section, OSS contributes to lymphatic vascular patterning and maturation, so iVECs model this biological process as well (Sweet et al. 2015). Using this model, we dissected the relationship between OSS, Wnt/β-catenin signaling, and FOXC2.

**Figure 5.**
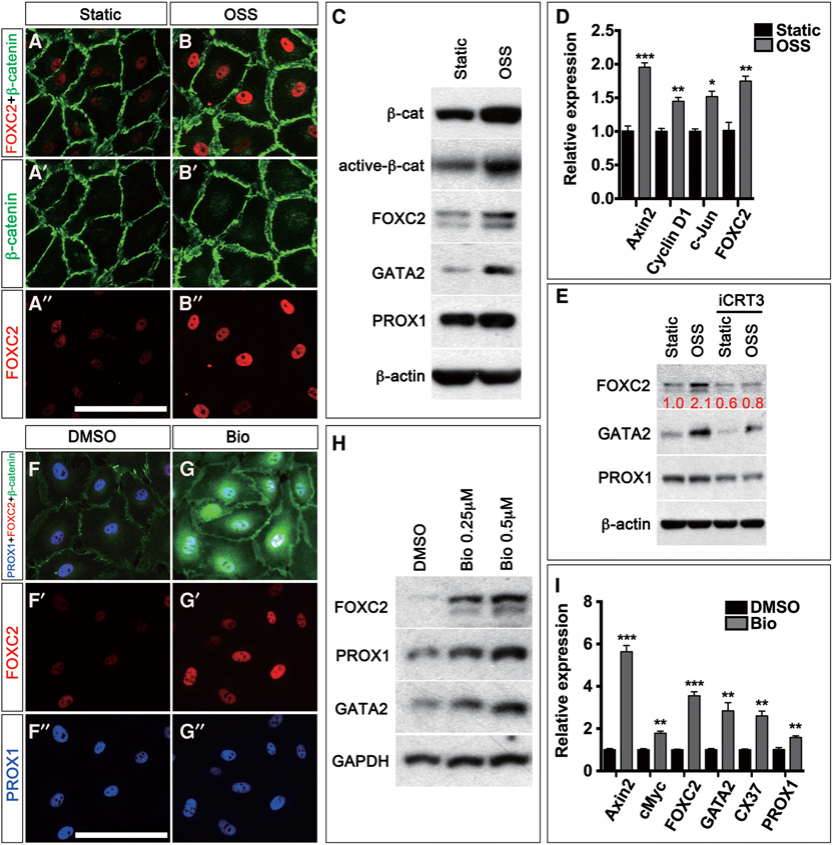
Wnt/β-catenin signaling is necessary and sufficient to regulate the expression of VEC markers in LECs. (*A*–*D*) Primary human LECs were cultured in the presence or absence of OSS for 48 h. Subsequently, cells were analyzed by IHC for the indicated markers (*A*,*B*), Western blot (*C*), or quantitative PCR (qPCR) (*D*). (*A*,*B*) IHC revealed the up-regulation of FOXC2 and β-catenin expression by OSS. (*C*) Western blot revealed an increase in the expression levels of total and active β-catenin. Valve-expressed transcription factors GATA2 and FOXC2 are also increased. PROX1 levels are not obviously changed. (*D*) qPCR validated the up-regulation of FOXC2. Additionally, target genes of the Wnt/β-catenin signaling pathway—Axin2, Cyclin-D1, and c-Jun—are increased. (*E*) LECs were cultured under static or OSS conditions in the presence or absence of 25 µM iCRT3, an antagonist of Wnt/β-catenin signaling, for 48 h. Western blot revealed a modest down-regulation of FOXC2, GATA2, and PROX1 expression by iCRT3 under static conditions. In contrast, iCRT3 dramatically inhibited the OSS-mediated up-regulation of FOXC2 and GATA2 expressions. The numbers in red indicate the relative expression of FOXC2 as measured densitometrically. (*F*–*I*) Primary human LECs were cultured in the presence of 0.5 µM Bio, an agonist of the Wnt/β-catenin signaling pathway, for 12 h. Subsequently, cells were analyzed by IHC for the indicated markers (*F*,*G*), Western blot (*H*), or qPCR (*I*). The results show that Bio enhances the expression of valve markers FOXC2, GATA2, PROX1, and CX37. Bars: *A*, *B*, *F*, *G*, 100 µm. *n* = 3 for each experiment. (*) *P* < 0.05; (**) *P* < 0.01; (***) *P* < 0.001.

OSS is known to activate the Wnt/β-catenin signaling pathway in blood ECs (Li et al. 2014; Kim et al. 2015). We wanted to determine whether this relationship is conserved in LECs. We exposed LECs to OSS by culturing them on a rocker within a sterile, humidified incubator. With this approach, we were able to generate iVECs with the same characteristic response to OSS as those generated using flow chambers. Specifically, we observed up-regulation of FOXC2, GATA2, membrane-bound β-catenin, VE-Cadherin, and nonphosphorylated YAP as originally reported (Fig. 5A–C; Kazenwadel et al. 2015; Sabine et al. 2015; Sweet et al. 2015; data not shown). PROX1 expression is not changed by OSS-mediated differentiation of LECs, as reported previously (Fig. 5C). Importantly, we observed an increase in the expression levels of total β-catenin and the hypophosphorylated active form of β-catenin in iVECs after OSS treatment (Fig. 5C). Wnt/β-catenin targets such as Axin2, Cyclin-D1, and cJun were also up-regulated by OSS (Fig. 5D). These results indicate that OSS activates Wnt/β-catenin signaling in LECs during the differentiation of LECs into iVECs.

To determine whether Wnt/β-catenin signaling is required to generate iVECs, we exposed LECs to OSS in the presence of iCRT3, a small molecule that inhibits the TCF/β-catenin interaction and abrogates Wnt/β-catenin signaling (Gonsalves et al. 2011). We found that iCRT3 treatment precluded the up-regulation of FOXC2 and GATA2 by OSS (Fig. 5E). PROX1 expression was modestly down-regulated by iCRT3, although it was not affected by OSS. These results indicate that Wnt/β-catenin signaling is necessary for OSS-mediated differentiation of LECs into iVECs.

Finally, we wanted to determine whether Wnt/β-catenin signaling is sufficient to induce the reprogramming of LECs into iVECs. We treated LECs with Bio, a chemical compound that inhibits GSK3β, stabilizes β-catenin, and activates Wnt/β-catenin signaling (Sato et al. 2004). As expected, Bio stabilized β-catenin in LECs and enhanced the expression of known targets of Wnt/β-catenin signaling such as Axin2 and cMyc (Fig. 5F,G,I). In addition, the expression of genes such as *FOXC2*, *GATA2*, and *CX37* was also enhanced by Bio without OSS treatment (Fig. 5F–I). PROX1 expression was also modestly enhanced by Bio. These results indicate that Wnt/β-catenin signaling is necessary and sufficient to promote the reprogramming of LECs into iVECs. Importantly, Wnt/β-catenin signaling is upstream of all known regulators of VEC fate.

### FOXC2 and PROX1 are direct targets of Wnt/β-catenin signaling

**Figure 6.**
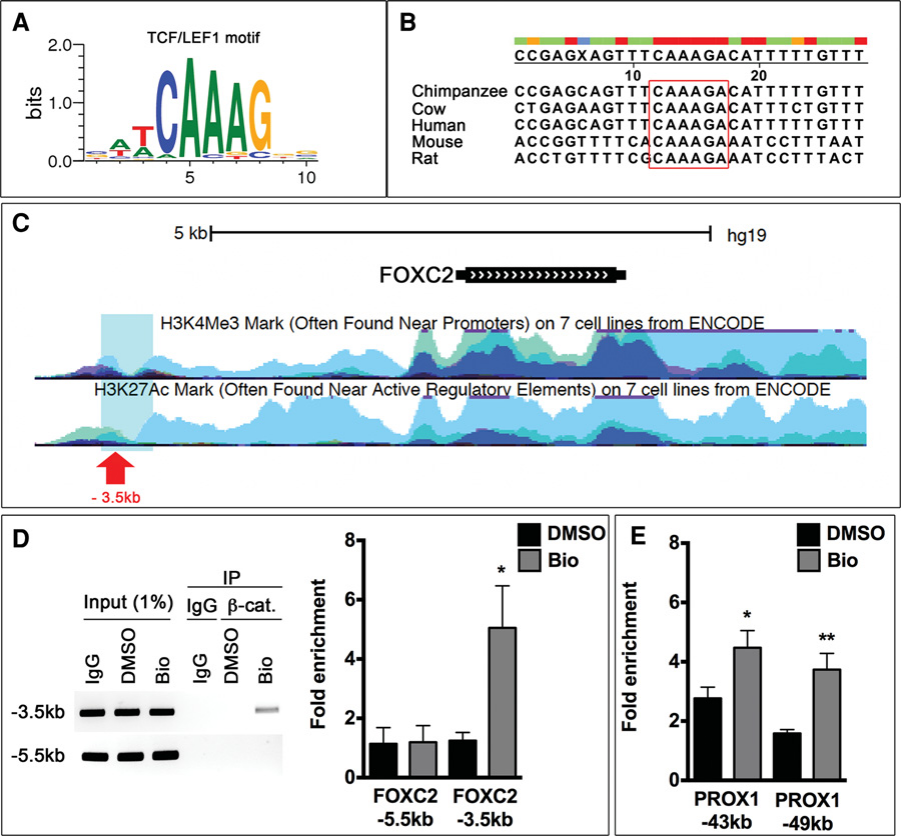
β-Catenin directly associates with the regulatory elements of *FOXC2* and *PROX1*. (*A*) TCF/LEF family transcription factors associate with the indicated motif. β-Catenin recognizes and binds this consensus sequence via TCF/LEF when Wnt/β-catenin signaling is active. (*B*) Genomic alignment was performed using the regulatory elements of the genes encoding FOXC2 from various mammals. Nucleotides in red are highly conserved. A highly conserved TCF/LEF-binding site is observed in the regulatory elements of *FOXC2* (enclosed within the red box). (*C*) The TCF/LEF-binding site (red arrow) is located ∼3.5 kb upstream of the TSS of *FOXC2*. Based on ChIP-seq data curated by The ENCODE Project Consortium (2012), the TCF/LEF-binding site is located in a region (blue box) that is enriched for H3K4me3 and H3K27ac histone modifications that are associated with transcriptionally active genes. (*D*) Primary human LECs were cultured in the presence of 0.5 µM DMSO or Bio for 12 h. Subsequently, cells were harvested, and ChIP was performed using IgG or an antibody specific to β-catenin. PCR was performed using primers flanking the TCF/LEF-binding site in the −3.5-kb location. As a negative control, primers flanking a TCF/LEF-binding site that is located at a more upstream location (−5.5 kb) were used. Gel and qPCR results show that β-catenin associates with the −3.5-kb site but not the −5.5-kb site in Bio-treated LECs. (*E*) β-Catenin associates with the TCF/LEF-binding sites in the regulatory elements of *PROX1* at the indicated locations. This interaction is significantly enhanced by Bio. (*D*,*E*) *n* = 3. (*) *P* < 0.05; (**) *P* < 0.01.

The sequence preference of TCF/LEF transcription factors is well characterized (Fig. 6A; Korinek et al. 1997). By using genomic alignments, we identified a highly conserved TCF/LEF-binding site in the regulatory element of *FOXC2*. This site is located ∼3.5 kb upstream of the transcription start site (TSS) (Fig. 6B). According to the ChIP-seq (chromatin immunoprecipitation [ChIP] combined with high-throughput sequencing) data curated by The ENCODE Project Consortium (2012), TCF4 (also known as TCF7L2) binds to this site in two cell lines. Furthermore, in seven cell lines, this TCF/LEF-binding site is marked with trimethylated H3K4 (H3K4me3) and acetylated H3K27 (H3K27ac), epigenetic modifications that are frequently associated with transcriptionally active regulatory elements (Fig. 6C). To determine whether β-catenin directly associates with this regulatory element in LECs, we treated LECs with the Wnt/β-catenin agonist Bio and performed ChIP. We found that β-catenin binding to the *FOXC2* regulatory element is significantly enhanced in Bio-treated LECs (Fig. 6D). In contrast, no significant enrichment is observed for a TCF/LEF-binding site that is located 5 kb upstream of the TSS of *FOXC2* in a region devoid of H3 epigenetic modifications.

We also evaluated the ability of β-catenin to interact with the regulatory elements of *PROX1*, which is a known target of the Wnt/β-catenin signaling pathway, in other contexts such as colon cancer cells and neural stem cells (Petrova et al. 2008; Karalay et al. 2011). Recently, Wnt/β-catenin signaling was also shown to promote PROX1 expression in human embryonic stem cells (Nicenboim et al. 2015). Two TCF/LEF-binding sites are located 43 and 49 kb upstream of the TSS of *PROX1*, and Wnt/β-catenin signaling enhances PROX1 expression via these sites (Petrova et al. 2008; Karalay et al. 2011).

Our ChIP assay revealed that the association of β-catenin with the regulatory elements upstream of *PROX1* is significantly enhanced in Bio-treated LECs (Fig. 6E). As a positive control, we confirmed that Bio promoted the association of β-catenin with the Wnt response element (WRE) of *AXIN2* in LECs (data not shown). These results show that Wnt/β-catenin signaling directly regulates PROX1 and FOXC2 expression in LECs.

### FOXC2 compensates for the loss of Wnt/β-catenin signaling in lymphatic vascular patterning

**Figure 7.**
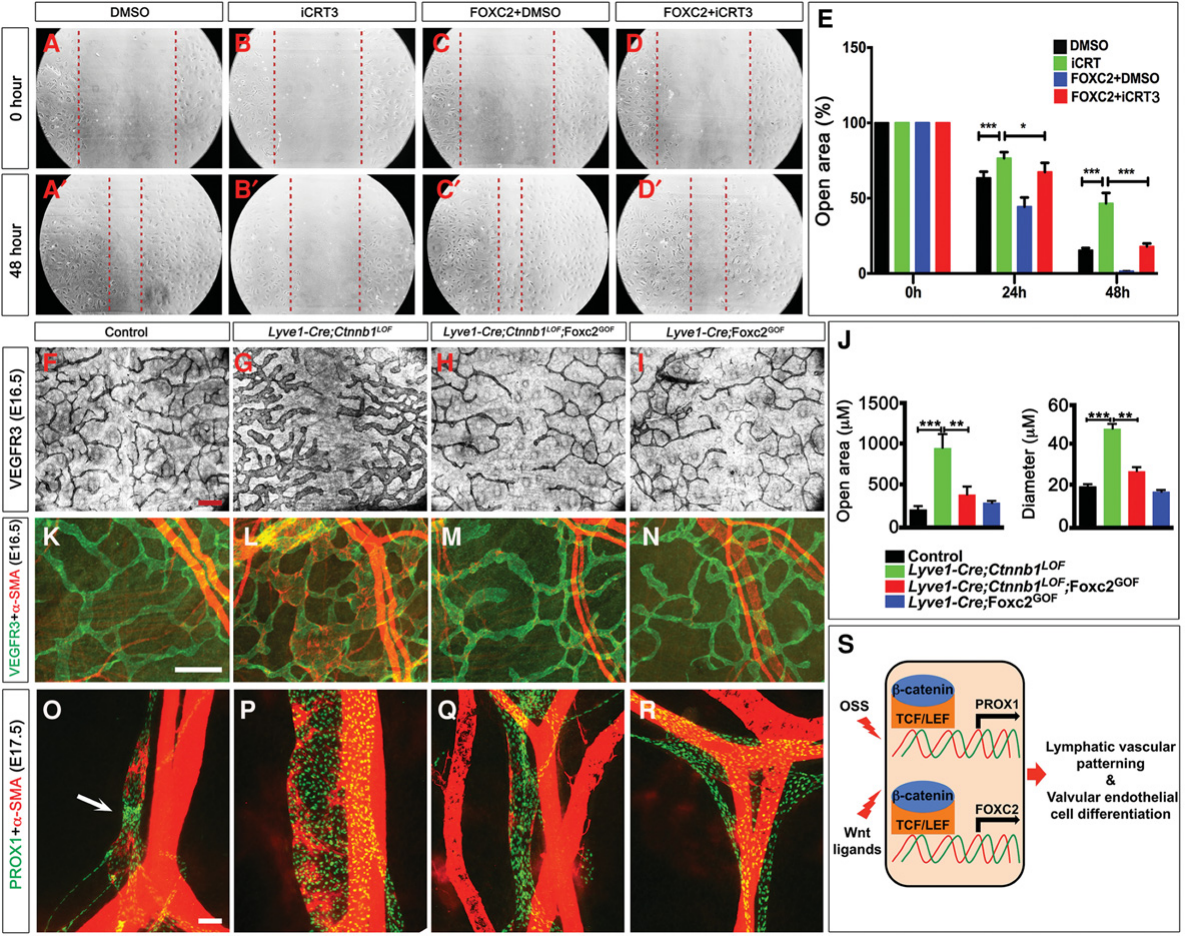
FOXC2 compensates for the loss of β-catenin to regulate lymphatic vessel patterning. (*A*–*E*) Primary human LECs were infected with control or FOXC2-expressing retroviral particles. Scratch assay was performed 24 h later in the presence or absence of 25 µM iCRT3. The space between the red dotted lines indicates the open scratch wound. (*E*) The wound size was measured at various time points and plotted. iCRT3 significantly inhibits the ability of control LECs to “heal” the scratch wound. FOXC2-overexpressing cells are able to significantly overcome iCRT3-induced inhibition. (*F*–*I*) The lymphatic vessels of the dorsal skin of E16.5 control (*F*), *Lyve1-Cre;Ctnnb1*^*LOF*^ (*G*), *Lyve1-Cre;Ctnnb1*^*LOF*^;FOXC2^GOF^ (*H*), and *Lyve1-Cre*;FOXC2^GOF^ (*I*) embryos were analyzed by IHC for VEGFR3. (*J*) The diameter of the vessels and the distance between the tip cells of the opposing fronts were quantified (Supplemental Fig. 7E–H) and plotted. In comparison with control embryos, the lymphatic vessels of *Lyve1-Cre;Cttnb1*^*LOF*^ embryos are significantly dilated. The distance between the migrating fronts is also significantly increased, indicating lymphatic vascular hypoplasia. Ectopic expression of FOXC2 significantly rescues these defects. (*K*–*N*) Coimmunohistochemistry for the indicated markers revealed the presence of α-SMA^+^ mural cells on the VEGFR3^+^ lymphatic vessels of *Lyve1-Cre;Cttnb1*^*LOF*^ embryos (shown in *L*). (*M*) This defect is fully rescued by ectopic expression of FOXC2. Lower magnification pictures of *F*–*I* and *K*–*N* are presented in Supplemental Figure 7. (*O*–*R*) The mesenteric lymphatic vessels of E17.5 embryos were analyzed by IHC for the indicated markers. Dilation of lymphatic vessels and the abnormal recruitment of α-SMA^+^ mural cells caused by the loss of β-catenin were rescued by FOXC2 overexpression. LVs are seen in control (arrow) but not in any of the mutant embryos. (*S*) Model for the relationship between OSS, β-catenin, FOXC2, and PROX1 during lymphatic vascular development. We did not exclude the role of yet to be identified Wnt ligands that function independently or in cooperation with OSS to activate β-catenin. Bars, 100 µm. (*A*–*E*) *n* = 3. (*F*–*R*) *n* = 4. (*) *P* < 0.05; (**) *P* < 0.01; (***) *P* < 0.001.

The defects in the tip cells and filopodia of mice lacking FOXC2 or β-catenin suggested that these molecules might be necessary for the migration of LECs. We evaluated this possibility by using an in vitro wound healing assay in which a scratch is introduced within a plate of confluent LECs and the time for cells to migrate and close the “wound” is evaluated. We found that the Wnt/β-catenin antagonist iCRT3 significantly delayed wound closure, suggesting an inhibition of LEC migration (Fig. 7A,B,E). An identical result was obtained when LECs were treated with XAV-939, another Wnt/β-catenin signaling antagonist (data not shown). These results support our hypothesis that the Wnt/β-catenin signaling pathway is critical for LEC migration.

In order to determine whether FOXC2 could compensate for the loss of Wnt/β-catenin signaling in cell migration, we repeated the scratch assay using LECs that are infected with retroviral particles to overexpress FOXC2 (Mani et al. 2007). We determined that FOXC2-overexpressing LECs migrated significantly faster than control cells (Fig. 7A,C,E). Furthermore, in the presence of iCRT3, FOXC2-overexpressing cells migrated faster than control cells (Fig. 7B,D,E). These results indicated that FOXC2 could compensate for the loss of the Wnt/β-catenin signaling pathway during the migration of LECs in vitro.

We next wanted to determine whether FOXC2 could compensate for the loss of Wnt/β-catenin signaling in vivo. We generated a transgenic mouse line that conditionally over-expresses FOXC2 using a previously reported approach (Berger et al. 2007). In this mouse line, a constitutively active regulatory element is upstream of an EGFP-3XpA cassette flanked by LoxP sites. The cDNA coding for FOXC2 is downstream from this cassette. Thus, FOXC2 is expressed only when the EGFP-3XpA cassette is deleted by Cre recombinase. We refer to this gain-of-function mouse line as FOXC2^GOF^.

We generated *Lyve1-Cre;Cttnb1*^*LOF*^;FOXC2^GOF^ embryos and found that they exhibit reduced edema compared with *Lyve1-Cre;Cttnb1*^*LOF*^ embryos (Supplemental Fig. 10B–E). The diameter of the lymphatic vessels and their migration pattern are comparable in control and *Lyve1-Cre*;FOXC2^GOF^ embryos (Fig. 7F–J; Supplemental Fig. 10F–H). Further analysis revealed that the morphological defects in the lymphatic vessels of E16.5 *Lyve1-Cre;Ctnnb1*^*LOF*^ embryos—namely, the dilation, growth retardation (Fig. 7F–J; Supplemental Fig. 10F–H), and abnormal recruitment of mural cells by the lymphatic capillaries (Fig. 7K–N; Supplemental Fig. 10I–L)—are completely rescued by overexpression of FOXC2. We also determined that the lymphatic vessels in the mesenteries of *Lyve1-Cre;Ctnnb1*^*LOF*^;FOXC2^GOF^ embryos have a normal morphology (Fig. 7O–R).

LVVs and VVs are present in E16.5 wild-type and *Lyve1-Cre*;FOXC2^GOF^ embryos but not in their *Lyve1-Cre;Ctnnb1*^*LOF*^ and *Lyve1-Cre;Cttnb1*^*LOF*^;FOXC2^GOF^ littermates (Supplemental Fig. 11). Therefore, FOXC2 is unable to compensate for the loss of β-catenin during LVV and VV development. Additional targets of Wnt/β-catenin signaling, such as PROX1, CX37, and GATA2, are likely necessary for the differentiation of VECs.

Intriguingly, LVs are absent, and PROX1 expression appears to be higher in the lymphatic vessels of *Lyve1-Cre*;FOXC2^GOF^ embryos (Fig. 7R). FOXC2 is expressed in a dynamic manner during the maturation of collecting lymphatic vessels (Norrmen et al. 2009). Specifically, after a sharp up-regulation at E15, FOXC2 expression is down-regulated before E16.5. PROX1 expression is down-regulated during this same time window (Norrmen et al. 2009). We speculate that the constant transgenic overexpression of FOXC2 prevents PROX1 down-regulation and collecting vessel maturation from happening normally.

In summary, these results indicate that FOXC2 could partially compensate for the loss of the Wnt/β-catenin signaling pathway in vivo and rescue the lymphatic vascular patterning defects of mice lacking β-catenin.

## Discussion

In this study, we identified the canonical Wnt/β-catenin signaling pathway as a critical regulator of valve development and the patterning of lymphatic vessels. Conditional deletion of β-catenin in *Lyve1-Cre* mice resulted in the complete absence of LVVs, LVs, and VVs. Additionally, lymphatic vessels are dilated, mispatterned, and abnormally covered by mural cells. These phenotypes are identical to that of *Foxc2*^−/−^ embryos. Consistently, FOXC2 compensates for the loss of β-catenin and rescues the lymphatic vascular patterning defects. However, FOXC2 is not sufficient to rescue the valve defects. Additional targets of the Wnt/β-catenin signaling pathway such as GATA2 and PROX1 are likely necessary for valve formation. Importantly, we revealed that OSS is the upstream activator of the Wnt/β-catenin signaling pathway. Indeed, activation of the Wnt/β-catenin signaling pathway is necessary for OSS-mediated up-regulation of critical genes such as FOXC2 and GATA2. We present a schematic of our model in Figure 7S.

### OSS and lymphatic vascular development

The pioneering work by Sabine et al. (2012) and Sweet et al. (2015) revealed the importance of fluid flow in lymphatic vascular development. These investigators exposed LECs to OSS of 0.5 dynes/cm^2^ in flow chambers to generate valve-like cells from LECs. We used a simple test tube rocker-based approach to generate iVECs. According to our calculations, LECs are exposed to an OSS that peaks at 0.2 dynes/cm^2^ for the six-well plates with a time average mean shear stress of 0 dynes/cm^2^ for all of the conditions (see the Materials and Methods for a detailed explanation of the analytical determination of wall shear stress). A similar result was obtained when the experiments were performed using 24-well plates with a shear stress of 0.09 dynes/cm^2^ (data not shown). Despite the apparent difference in the shear stress values, we were able to precisely recapitulate the results of Sabine et al. (2012). This observation has interesting implications regarding the mechanisms of iVEC differentiation. The OSS value used by Sabine et al. (2012) was similar to values measured in the mesenteric lymphatic vessels of adult rats (Dixon et al. 2006). Due to technical limitations, we currently do not know the OSS values in the developing lymphatic vessels of mice. However, given the apparent conservation in the mechanisms, we reason that the flow pattern rather than the absolute shear stress value is important for lymphatic vascular development. Our in vitro data are consistent with this hypothesis. Furthermore, our finding provides an inexpensive and easily accessible approach that will accelerate the field of lymphatic research.

### OSS and PROX1 expression

OSS was originally proposed to be critical for LV development (Sabine et al. 2012). Although PROX1 is strongly expressed in the VECs, its expression is not promoted by OSS. However, the Wnt agonist Bio promotes PROX1 expression in LECs (Fig. 4). Why does activation of the Wnt/β-catenin signaling pathway but not OSS have the capacity to promote PROX1 expression in LECs? Signaling pathways activate their target genes in a signal intensity-dependent and time-dependent manner (Wolpert 2016). Therefore, it is conceivable that the strength of the Wnt/β-catenin signaling pathway activated by OSS in vitro is insufficient to activate PROX1 expression. The three-dimensional architecture of the valve-forming regions likely enhances and sustains OSS-mediated signals in vivo. Valves are composed of upstream and downstream portions with distinct molecular profiles (Kanady et al. 2011; Sabine et al. 2012; Munger et al. 2013). The “OSS model” recapitulates the cells on the downstream side of valves by up-regulating markers such as FOXC2 and CX37. It is possible that the cells on the upstream side of the valve, which are missing in this assay, might be necessary to promote PROX1 expression and valve morphogenesis. Consistent with this possibility, whereas VECs have an elongated architecture in vivo, iVECs are cuboidal (Sabine et al. 2012; Tatin et al. 2013; Geng et al. 2016). Cell–cell and cell–matrix interactions between the upstream and downstream sides of the valves could modulate the flow-mediated mechanical force experienced by the cells. Alternatively, the cells located in the upstream side of the valves might provide signals to modulate OSS and the Wnt/β-catenin signaling pathway. In mammals, there are 19 Wnt ligands that are frequently coexpressed and could compensate for each other (Logan and Nusse 2004). At least five Wnt ligands (Wnt2, Wnt3a, Wnt4, Wnt7b, and Wnt9b) are expressed in the cardiac valves where the Wnt/β-catenin signaling pathway is known to be necessary for cardiac cushion formation and maturation (Alfieri et al. 2010). Whether these ligands play any role during lymphatic vessel growth and valve formation remains to be investigated.

### OSS, lymphatic vascular maturation, and valve development

Our results show that OSS promotes Wnt/β-catenin signaling and FOXC2 expression, both of which are necessary in turn for valve formation and lymphatic vascular maturation. While FOXC2 compensates for the loss of β-catenin in lymphatic vessels, other targets of Wnt/β-catenin signaling appear to be necessary for valve formation. What are the factors that modulate the LEC response to OSS and determine whether they differentiate into valves or mature lymphatic vessels? We speculate that the OSS response could be regulated by whether PROX1 is up-regulated at a specific site. Accordingly, OSS and OSS+PROX1 will promote lymphatic vascular maturation and valve formation, respectively.

In the future, it will be important to determine the OSS-specific effects on LECs. Valve-forming cells undergo complex migratory events such as delamination, realignment with respect to flow, condensation, and elongation to form valves (Geng et al. 2016). Lymphatic vascular patterning involves the growth of the lymphatic plexus (Coxam et al. 2014). Therefore, cell migration might be the cellular event that is conserved between these two processes. Our work has revealed that the tip cells, which are important for cell migration, are defective in mice lacking FOXC2. We also provided evidence that FOXC2 is a target of the Wnt/β-catenin signaling pathway during lymphatic vascular development. It will be important to mechanistically dissect the relationship between FOXC2 and cell migration.

### Sensing OSS

Despite its elegance, a definitive proof for the accuracy of the “OSS model” is still missing. Identifying the mechanosensory molecules in the lymphatic vasculature will bring us a step closer to testing this model in vivo. Primary cilia function as mechanosensors in many cell types (Ingber 2006). Using SEM, we failed to observe any primary cilia in the developing LVV-ECs (Supplemental Fig. 12). Based on the published literature, we discuss a few alternate mechanisms that may be involved in sensing and translating OSS into Wnt/β-catenin signals.

#### Integrins

Integrins are well-known mechanosensors in numerous contexts (Ingber 2006). Integrin α9 and integrin α5 are both critical for LVV and LV development (Bazigou et al. 2009; Turner et al. 2014). Integrin α5β1 was shown recently to enhance Wnt/β-catenin signals in osteoblasts via the PI3K–AKT pathway (Saidak et al. 2015).

#### Wnt receptors

Frizzled receptors might function as mechanosensors, as reported recently (Rotherham and El Haj 2015). According to this model, shear activated Frizzled receptors could interact with LRP5/6 and activate the downstream signals.

#### Receptor tyrosine kinases

In tumor cells, mechanical strain activates a receptor tyrosine kinase (Ret) that phosphorylates and promotes the nuclear translocation of β-catenin by inhibiting catenin–cadherin interaction at cell junctions (Fernandez-Sanchez et al. 2015). VEGFR2 and VEGFR3 are mechanoresponsive and may play the role of Ret in ECs (Coon et al. 2015).

#### Ion channels

Mutations in the mechanically activated ion channel PIEZO1 are associated with human lymphedema (Fotiou et al. 2015; Lukacs et al. 2015). Hence, PIEZO1 and its paralog, PIEZO2, might be involved in sensing OSS.

### Evolutionarily divergent roles of the Wnt/β-catenin signaling pathway in lymphatic vascular development

The Wnt/β-catenin signaling pathway was shown recently to promote PROX1 expression and asymmetric division of specialized angioblast cells in the venous niche of zebrafish. These cells give rise to the lymphatic vessels of zebrafish (Nicenboim et al. 2015). As shown above, conditional deletion of β-catenin using *Lyve1-Cre* in mice did not reveal any obvious defects in LEC progenitor specification. Conditional deletion of β-catenin using Tie2-Cre in the entire vascular network resulted in embryonic death at E12.5, as reported previously (Cattelino et al. 2003). Analysis of these mutants did not reveal any obvious lymphatic vascular defects (data not shown). PROX1^+^ LECs appear to be normally specified in these mutants.

Despite numerous similarities, some important differences between mammalian and zebrafish lymphatics exist. Relevant to this work, the lymphatic vasculature of zebrafish is devoid of LVs (Koltowska et al. 2013). Furthermore, the site of lymph return to blood circulation is currently unknown in these animals. Therefore, it is possible that Wnt/ β-catenin signaling pathway is playing species-specific roles in lymphatic vascular development.

### Clinical implications

Approaches to treat lymphedema currently do not exist. We identified an important signaling pathway that regulates lymphatic vascular morphogenesis. Numerous agonists and antagonists of the Wnt/ β-catenin signaling pathway are currently available. Some of these molecules are in clinical trials for various diseases such as cancer and Alzheimer's disease (MacDonald et al. 2009). A better understanding of the spatial and temporal regulations of the Wnt/ β-catenin signaling pathway within the lymphatic vasculature will provide exciting opportunities to treat lymphedema, potentially by repurposing existing drugs.

## Materials and methods

### Mouse models

TCF/LEF-H2B-EGFP, *Lyve1-Cre*, *Ctnnb1*^+/*f*^, ProxTom, *Prox1*^+/*Cre*^, *Foxc2*^+/−^, and *Clec2*^−/−^ mice were reported previously (Brault et al. 2001; Bertozzi et al. 2010; Ferrer-Vaquer et al. 2010; Pham et al. 2010; Srinivasan et al. 2010; Truman et al. 2012; Geng et al. 2016). We generated the FOXC2^GOF^ mouse line by inserting a cDNA coding for *Foxc2* into the JOJO plasmid (Berger et al. 2007). Linearized plasmid was electroporated into blastocysts, and the transgenic founders were visually selected based on the expression of EGFP. All mice were housed and handled according to the Institutional Animal Care and Use Committee protocols.

### Antibodies

Primary antibodies used for immunostaining were as follows: rabbit anti-PROX1 (AngioBio), goat anti-human PROX1 (R&D Systems), sheep anti-mouse FOXC2 (R&D Systems), goat anti-mouse VEGFR3 (R&D Systems), goat anti-mouse LYVE1 (R&D Systems), rat anti-mouse CD31 (BD Pharmingen), goat anti-mouse GATA2 (R&D Systems), rabbit anti-total β-catenin and rabbit anti-active β-catenin antibodies (both from Cell Signaling Technologies), goat anti-mouse NRP2 (R&D Systems), chicken anti-GFP (Abcam), rat anti-mouse Endomucin (eBioscience), rabbit anti-CX37 (Invitrogen), and Cy3-conjugated monoclonal anti-α-SMA (Sigma-Aldrich). The following secondary antibodies were used: Cy3-conjugated donkey anti-rabbit, Cy3-conjugated donkey anti-sheep, and Cy5-conjugated donkey anti-rat (all purchased from Jackson ImmunoResearch Laboratories). Alexa 488-conjugated donkey anti-goat, Alexa 488-conjugated goat anti-chicken, and Alexa 488-conjugated donkey anti-rat were purchased from Life Technologies.

Primary antibodies used for Western blotting were as follows: mouse anti- β-Actin (Sigma), mouse anti-mouse β-catenin (BD Pharmingen), rabbit anti-active β-catenin (Cell Signaling Technologies), goat anti-human PROX1 (R&D Systems), sheep anti-mouse FOXC2 (R&D Systems), and goat anti-mouse GATA2 (R&D Systems). The following HRP-conjugated secondary antibodies from Santa Cruz Biotechnology were used: goat anti-mouse IgG, goat anti-rabbit IgG, donkey anti-goat IgG, and donkey anti-sheep IgG.

### IHC

IHC on sections was done according to our detailed protocols that we published recently (Geng et al. 2016). Whole-mount IHC using the skin, gut, or heart was performed using a modified iDISCO protocol (Renier et al. 2014). Briefly, isolated samples were fixed in 4% PFA for 4 h at 4°C and washed profusely with PBST (0.2% Triton X-100 in PBS). The samples were incubated in PBSTD (0.2% Triton X-100, 20% DMSO in PBS) overnight and incubated in PBSTTDND (0.1% Tween X-100, 0.1% Triton X-100, 0.1% deoxycholate, 0.1% NP40, 20% DMSO in PBS) overnight at room temperature. Pretreated samples were incubated with PBSTDM (0.2% Triton X-100, 20% DMSO, 0.3 M glycine in PBS) overnight and blocked with PBSTD (0.2% Triton X-100/10% DMSO/6% donkey serum in PBS) overnight. Samples were incubated overnight with primary antibodies diluted in PTwHD (0.2% Tween-20, 10 µg/mL heparin, 10% DMSO, 3% donkey serum in PBS). After profuse washing, samples were incubated overnight with secondary antibodies diluted in PTwH (0.2% Tween-20, 10 µg/mL heparin, 3% donkey serum in PBS). A lighter fixation protocol (1% PFA for 1 h at 4°C) was used for whole-mount IHC with anti CX37 antibody. Samples were visualized and analyzed as described previously (Geng et al. 2016).

### In situ hybridization

In situ hybridization and coimmunohistochemistry experiments were performed as described previously (Srinivasan et al. 2010). Information regarding the *Axin2* probe will be provided on request.

### Western blot

Cells were lysed in lysis buffer (20 mM Tris–HCl at pH 7.5, 150 mM NaCl, 1.0% Triton X-100, 20 mM NaF, 2 mM EDTA, 2 mM Na-orthovanadate, 1 mM phenylmethylsulfonyl fluoride [PMSF], 5 mg/mL leupeptin A). Concentration of proteins was measured using Bradford reagent (Bio-Rad). Western blot was carried out according to standard protocols.

### SEM

SEM was done according to our previous protocol (Geng et al. 2016).

### RNA isolation and quantitative real-time PCR

Total RNA was isolated from cells using Trizol reagent (Invitrogen) according to the manufacturer's protocol. cDNA was synthesized from total RNA using the SuperScript III first strand synthesis system (Invitrogen) following the manufacturer's instructions. Quantitative PCR (qPCR) was performed using SYBR Green PCR master mix reagent (Bio-Rad) in a CFX96 real-time system (Bio-Rad). Primer sequences will be provided on request. The threshold cycle (Ct) value for each gene was normalized to the Ct value for β-actin.

### ChIP

ChIP assays were performed using 8 × 10^6^ to 10 × 10^6^ human primary LECs (Lonza) as described previously (Kim et al. 2005). Cells at 90% confluency were treated with 0.5 µM DMSO or Bio for 12 h. Subsequently, LECs were fixed in 1% formaldehyde for 30 min at room temperature, and glycine at a final concentration of 0.125 M was added for 10 min. Cells were washed, harvested with cold PBS, resuspended in lysis buffer 1 (50 mM Hepes-KOH at pH 7.5, 140mM NaCl, 1.0 µM EDTA, 10% glycerol, 0.5% NP-40, 0.25% Triton X-100, protease inhibitor cocktail), and rotated for 5 min at 4°C. Cells were spun down at 2000 rpm for 3 min, and supernatant was removed. The cell pellet was resuspended in lysis buffer 2 (200 mM NaCl, 1.0 µM EDTA, 0.5 µM EGTA, 10 µM Tris-HCl at pH 8, protease inhibitor cocktail) for 5 min at 4°C with rotation. After centrifugation at 2000 rpm for 3 min, the supernatant was removed by aspiration. The pellet was resuspended using lysis buffer 3 (1.0 µM EDTA, 0.5 µM EGTA, 10 µM Tris-HCl at pH 8, protease inhibitor cocktail), sonicated on ice, and centrifuged at 13,000 rpm for 10 min. The supernatant was diluted in immunoprecipitation dilution buffer (20 mM Tris-HCl at pH 8.0, 150 mM NaCl, 1 mM EDTA, 1% Triton X-100, protease inhibitors) and used for downstream analysis. The cross-linked protein–DNA complexes were immunoprecipitated using 3.0 µg of mouse anti-β-catenin antibody (BD Pharmingen) or 1.0 µg of mouse IgG antibody (Santa Cruz Biotechnology). qPCR was performed as described above using primers flanking the predicted TCF/LEF sites or control sites. Primer sequences will be provided on request.

### Wound healing assay

Primary human LECs were plated in 24-well plates and infected with retroviral particles expressing control GFP or human FOXC2. When cells were near confluency (>90% density), scratch wounds were made at the center of the well using a sterile 1000-µL pipette tip. Loose cells were removed by a PBS wash, and DMSO-containing (Sigma) or iCRT3-containing (Sigma) medium was added. Images were recorded at *t* = 0, *t* = 24, and *t* = 48 h using an inverted microscope to measure the wound size.

### OSS

Human LECs were cultured to confluency in six-well plates and exposed to OSS using a test tube rocker (Thermolyne Speci-Mix aliquot mixer model M71015, Barnstead International) with a preset frequency (18 rpm). The entire setup was kept inside a sterile humidified incubator with 5% CO_2_ for 48 h. The rocker went through an angle of 48^o^ in ∼2 sec. One milliliter of medium was used for the 24-well plates, and 6 mL of medium was used for the six-well plates. In both the cases, the amount of medium was sufficient to cover the center of the wells at all times, although the medium did lose contact with the sides of the wells at high angles of oscillation.

An analytical formulation for a rectangular Petri dish was presented in Zhou et al. (2010), where the investigators derived the shear stress at the bottom of the dish as a function of time and position. The derivation involved the use of lubrication approximation, which is justified when low volumes of fluids are used compared with the cross-sectional area and assumed no slip boundary condition at the bottom of the dish and 0 velocity gradient at the free surface. With these conditions, the wall shear stress at the bottom of the well can be described by the equation

$$\begin{array}{rl} & |\tau_{w}|=\frac{3\pi \mu \theta_{max}x(L-x)}{T{\left[h_{0}cot\theta +\frac{L}{2}-x\right]}^{2}\sin^{2}\theta }\cos\frac{2\pi t}{T},\theta \leq \theta_{0}\\ & =\frac{3\pi \mu \theta_{max}x}{T{\left[\sqrt{2h_{0}Lcot\theta }-x\right]}^{2}\sin^{2}\theta }\cos\frac{2\pi t}{T},\theta \leq \theta_{0} \end{array},$$

where µ is the dynamic viscosity of the fluid, *L* is the length of the dish along the direction of motion of the rocker, *x* is the distance of the point of interest from a side of the dish, *h*_0_ is the height of the fluid surface when the dish is horizontal, θ is the angle of the rocker, θ_max_ is the maximum angle of the rocker, θ_0_ is the “critical flip angle” (defined as the angle at which the fluid just leaves contact with the bottom of the dish), *t* is the time, and *T* is the time period of the sinusoidal motion of the rocker.

The aforementioned analytical formulation was adapted for the present scenario, since the lubrication approximation holds for the small volume of fluid used, and the region of interest is the center of the wells, which was always covered with fluid. For the medium, µ is considered as the same as that of water, which is 1 centiPoise. The rocker had a θ_max_ of 48^o^ and *T* of ∼2 sec. The dish had an *L* of ∼3.48 cm for the six-well plates and 1.56 cm for the 24-well plates. The volume of fluid used was 6 mL and 1 mL, and the *h*_0_ was ∼0.63 cm for the six-well plates and ∼0.53 cm for the 24-well plates, respectively. With these values, the maximum shear stress is calculated to be ∼0.3 dynes/cm^2^ for the six-well plates and ∼0.09 dynes/cm^2^ for the 24-well plates at the center of the wells.

## Supplementary Material

Supplemental Material
