# Supplementary material for: Mechanotransduction activates canonical Wnt/β-catenin signaling to promote lymphatic vascular patterning and the development of lymphatic and lymphovenous valves
Source: Genes Dev. 2016 Jun 15;30(12):1454–69. doi: 10.1101/gad.282400.116 (PMC4926867; doi:10.1101/gad.282400.116)
Supplement: Supplemental Material [file supp_30_12_1454__index.html]

Mechanotransduction activates canonical Wnt/β-catenin signaling to promote lymphatic vascular patterning and the development of lymphatic and lymphovenous valves — Supplemental Material 

# Mechanotransduction activates canonical Wnt/β-catenin signaling to promote lymphatic vascular patterning and the development of lymphatic and lymphovenous valves

## Supplemental Material

**Files in this Data Supplement:**

- Supplemental\_FigureLegends.docx
- Supp\_Figure1.tif
- Supp\_Figure2.tif
- Supp\_Figure3.tif
- Supp\_Figure4.tif
- Supp\_Figure5.tif
- Supp\_Figure6.tif
- Supp\_Figure7.tif
- Supp\_Figure8.tif
- Supp\_Figure9.tif
- Supp\_Figure10.tif
- Supp\_Figure11.tif
- Supp\_Figure12.tif
