# Supplementary material for: Mechanotransduction activates canonical Wnt/β-catenin signaling to promote lymphatic vascular patterning and the development of lymphatic and lymphovenous valves
Source: Genes Dev. 2016 Jun 15;30(12):1454–69. doi: 10.1101/gad.282400.116 (PMC4926867; doi:10.1101/gad.282400.116)
Supplement: Supplemental Material [file supp_gad.282400.116_Supplemental_FigureLegends.docx]

**SUPPLEMENTARY FIGURE LEGENDS**

**Supplementary Figure 1: Wnt/β-catenin signaling pathway is active in LEC progenitors, VV-ECs and LV-ECs.**

TCF/LEF-H2BEGFP embryos were harvested at the indicated developmental- time points and analyzed for the expression of EGFP together with PROX1 and CD31 by immunohistochemistry. (A) At E11.0 EGFP signal is observed in a subset of LEC progenitors (arrows). Fewer LECs that are outside the CV are EGFP^+^. (B) At E15.5 strong EGFP expression is observed in the developing VV-ECs (arrowheads). (C) At E16.5 the newly differentiating PROX1^high^ LV-ECs of mesenteric lymphatic vessels are EGFP^+^ (arrows). (D) At E17.5 LV-ECs reorganize into a circular sheath. These cells are EGFP^+^ (arrows).

Abbreviations: CV, cardinal vein; A, aorta; LS, lymph sac; IJV, internal jugular vein, SCV, subclavian vein; SVC, superior vena cava.

Measuring bar: 100 μm for A-A’’; 100 μm for B-B’’; 100 μm for C-D’’

Statistics: n=4 for each stage.

**Supplementary Figure 2: β-catenin is necessary for proper lymphatic vascular development.**

(A-C) E14.5 *Lyve1-Cre;Ctnnb1^LOF^* embryos display edema (B, arrow) and rarely blood-filled lymphatic vessels in the peripheral skin (C, arrow).

(D-F) The lymph sacs of E14.5 *Lyve1-Cre;Ctnnb1^LOF^* embryos are significantly larger than that of control littermates, as revealed by immunohistochemistry for the indicated markers (D, E) and quantification (F).

Abbreviations: LS, lymph sac; IJV, internal jugular vein.

Measuring bar: 200 μm for D and E

Statistics: n=18 for A-C; n=4 for D and E; **=p<0.01.

**Supplementary Figure 3: LVV-EC differentiation does not occur in embryos lacking β-catenin.**

(A-D) 10 μm frontal sections prepared from E12.0 control (A, C) and *Lyve1-Cre;Ctnnb1^LOF^* (B, D) embryos were analyzed by immunohistochemistry using the specified valve-markers. While PROX1^high^ FOXC2^high^ GATA2^high^ LVV-ECs are observed in control embryos (A, C, arrows), they are absent in mutants (B, D, arrows).

(E, F) 500 μm sagittal sections were made from E12.0 ProxTom (E) or ProxTom; *Lyve1-Cre;Ctnnb1^LOF^* (F) embryos. Following whole mount immunohistochemistry for VEGFR3 and CD31 the samples were analyzed by confocal microscopy. Two clusters of LVV-ECs are observed in control embryos (E, arrows). But, these cells are absent in mutant embryos lacking β-catenin (F). (E’-F’’) The above samples were re-processed and SEM was performed. One of the LVV-EC clusters of the control embryo is in the field of view (E’, red dotted box). At higher magnification LVV-ECs are seen delaminating from the veins in multiple layers (E’’, magenta). (F’ and F’’) In contrast, in a corresponding region from embryos lacking β-catenin, the endothelial cells have a quiescent, cobblestone-like morphology.

(G, H) Model indicates that the differentiation of LVV-ECs (red cells) is defective in embryos lacking β-catenin. LECs (green cells) of control and mutant embryos are morphologically indistinguishable at this stage.

Abbreviations: LS, lymph sac; IJV, internal jugular vein; EJV, external jugular vein; SVC, superior vena cava; A, artery.

Measuring bar: 100 μm for A-D; 200 μm for E’ and F’; 50 μm for E, F, E’’, F’’

Statistics: n=4 for each stage.

**Supplementary Figure 4: LV-EC differentiation does not occur in embryos lacking β-catenin.**

(A, B) The mesenteric lymphatic vessels E17.5 Control (A) or *Lyve1-Cre;Ctnnb1^LOF^* (B) embryos were analyzed by whole mount immunohistochemistry for the indicated markers. LVs seen in control samples (A, arrows) are absent in mutants (B). Further, the lymphatic vessels of mutants are dramatically dilated. The number of lymphatic valves was quantified in panel C.

(D-G) The mesenteric lymphatic vessels of E16.5 and E17.5 control and *Lyve1-Cre;Ctnnb1^LOF^* embryos were analyzed. LV-EC differentiation begins with the upregulation of FOXC2, GATA2 and PROX1 expressions in the lymphatic vessels of controls (D, F, arrows). In contrast, LV-EC differentiation does not happen in mutants (E, G).

Measuring bar: 500 μm for A and B; 200 μm for D-E’; 200 μm for F-G’.

Statistics: n=4 for each stage; ***=p<0.001.

**Supplementary Figure 5: β-catenin is necessary for the patterning of lymphatic vessels in heart.**

(A, B) The lymphatic vessels in the hearts of E17.5 *Lyve1-Cre;Ctnnb1^LOF^* and its control littermates were analyzed by whole mount immunohistochemistry. The insets show lower magnification pictures of the heart. The lymphatic vessels of the controls have migrated until the posterior tip of the heart. In contrast, the lymphatic vessels of mutants have migrated only until the middle of the heart. (B, C) The lymphatic vessels of mutants are mispatterned and dilated. The diameter of vessels is quantified in C.

Measuring bar: 100 μm for A and B

Statistics: n=4 for each stage; **=p<0.01.

**Supplementary Figure 6: LEC identity is not compromised in mice lacking β-catenin.**

E14.5 *Lyve1-Cre;Ctnnb1^LOF^* and its control littermates were analyzed for the expression of endomucin (A) Endomucin is strongly expressed in the venous endothelial cells, but not in the LVV-ECs (A, arrows) or LECs of control embryos. (B) LVVs are absent in *Lyve1-Cre;Ctnnb1^LOF^* embryos (arrowheads). Endomucin is expressed in venous endothelial cells and in areas where LVVs would have normally formed. However, endomucin expression is not observed in the LECs of the lymph sacs (LS). (C, D) Whole mount immunohistochemistry revealed that endomucin is not expressed in the lymphatic vessels of control or mutant embryos.

Measuring bar: 100 μm for A and B; 200 μm for C and D

Statistics: n=3 for each stage.

**Supplementary Figure 7: PROX1 expression is affected in the lymphatic vasculature of mice lacking β-catenin.**

The lymphatic vessels of *Lyve1-Cre;Ctnnb1^LOF^* and its control littermates were analyzed by whole mount immunohistochemistry at the indicated stages. After capturing the images at identical conditions, the fluorescent intensity was measured in a semi-quantitative manner. PROX1 expression is strongest in LV-ECs, which are absent in *Lyve1-Cre;Ctnnb1^LOF^* embryos. PROX1 expression is modestly downregulated in the tip cells of mice lacking β-catenin. In contrast, PROX1 expression is upregulated in the collecting lymphatic vessels.

Statistics: n=3 for each stage; *=p<0.05; **=p<0.01; ***=p<0.001.

**Supplementary Figure 8: Expression of CX37 is dramatically downregulated in the LECs of mice lacking β-catenin.**

CX37 is expressed in the NRP2^+^ LECs of E15.5 control (A) but not *Lyve1-Cre;Ctnnb1^LOF^* embryos (B). CX37 expression in the arterial endothelial cells is not affected by the loss of β-catenin in LECs (arrows).

Measuring bar: 200 μm

Statistics: n=2.

**Supplementary Figure 9: Lymphatic vessels in the dorsal skin of *Clec2^-/-^* embryos are mispatterned.**

(A-B) Dorsal skins of E15.5 control and *Clec2^-/-^* littermates were analyzed using a VEGFR3-specific antibody. The magenta colored lines indicate the distance between the tip cells from the opposing front. This distance is dramatically increased in *Clec2^-/-^* embryos. Higher magnification pictures of the boxed regions are presented in A’ and B’ indicating that the lymphatic vessels in *Clec2^-/-^* are dilated and have an abnormal bulbous shape.

Measuring bar: 1000 μm for A and B; 200 μm for A’ and B’

Statistics: n=2.

**Supplementary Figure 10: FOXC2 rescues the lymphatic vascular defects of mice lacking β-catenin.**

(A-D) E14.5 *Lyve1-Cre;Ctnnb1^LOF^* embryos are severely edematous (B). In contrast *Lyve1-Cre;Ctnnb1^LOF^*;FOXC2^GOF^ littermates have dramatically reduced swelling in the peripheral skin (C). Control (A) and *Lyve1-Cre;*FOXC2^GOF^ (D) are morphologically indistinguishable.

(E-L) Peripheral skins of E16.5 embryos of the indicated genotypes were analyzed using the indicated markers. Higher magnification pictures of the boxed regions are presented in Figure 6. Magenta colored lines indicate the distance between the opposing migrating fronts. These distances were measured and plotted in Figure 6J. FOXC2 dramatically rescues the lymphatic vascular defects (distance between tips, vessel diameter and mural cell recruitment) in mice lacking β-catenin.

Measuring bar: 500 μm for E-H; 500 μm for I-L.

Statistics: n=4 for each stage.
