# Supplementary figures and images for: Mechanotransduction activates canonical Wnt/β-catenin signaling to promote lymphatic vascular patterning and the development of lymphatic and lymphovenous valves
Source: Genes Dev. 2016 Jun 15;30(12):1454–69. doi: 10.1101/gad.282400.116 (PMC4926867; doi:10.1101/gad.282400.116)

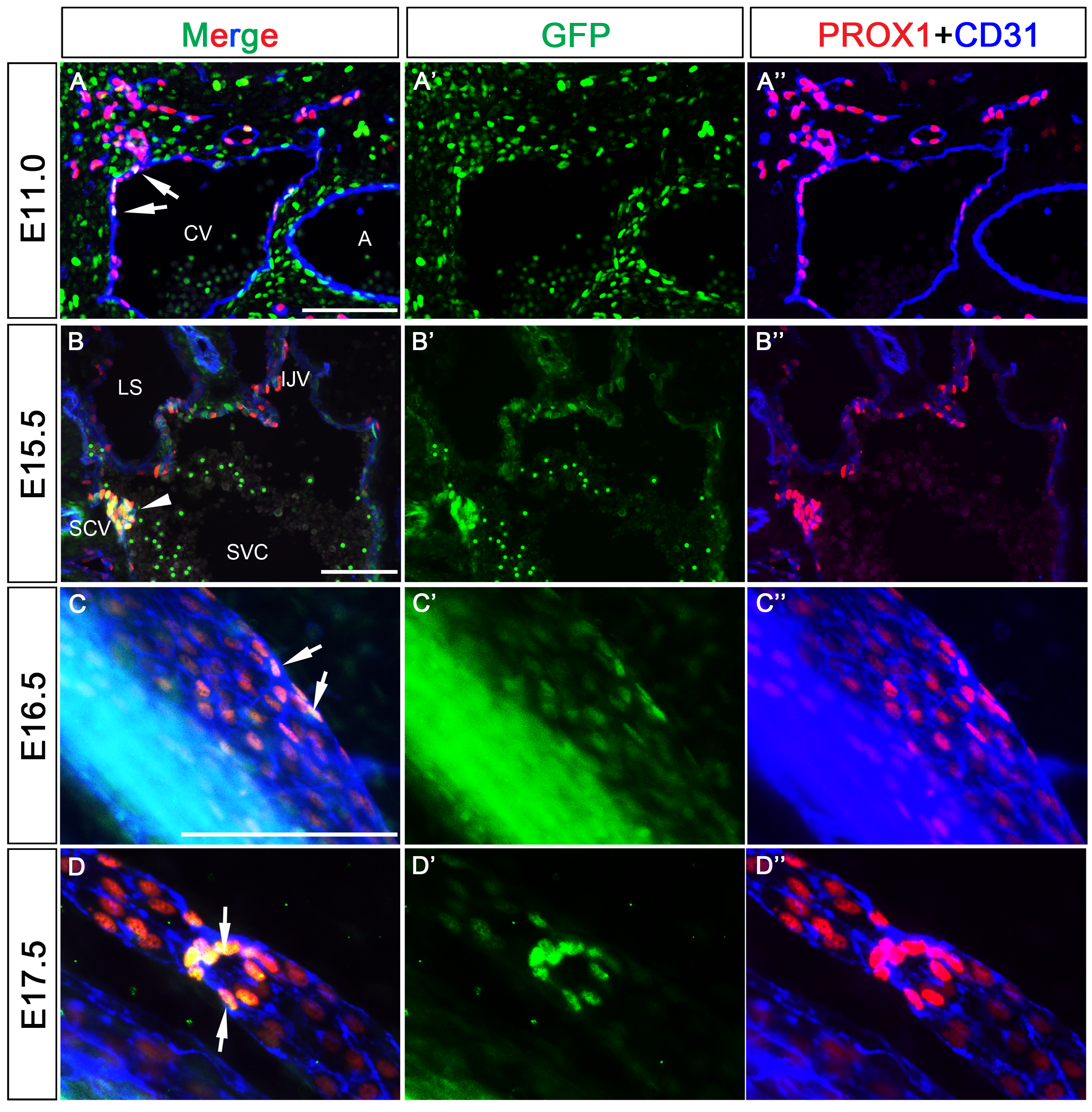

Supplement: Supplemental Material [file supp_gad.282400.116_Supp1.tif]

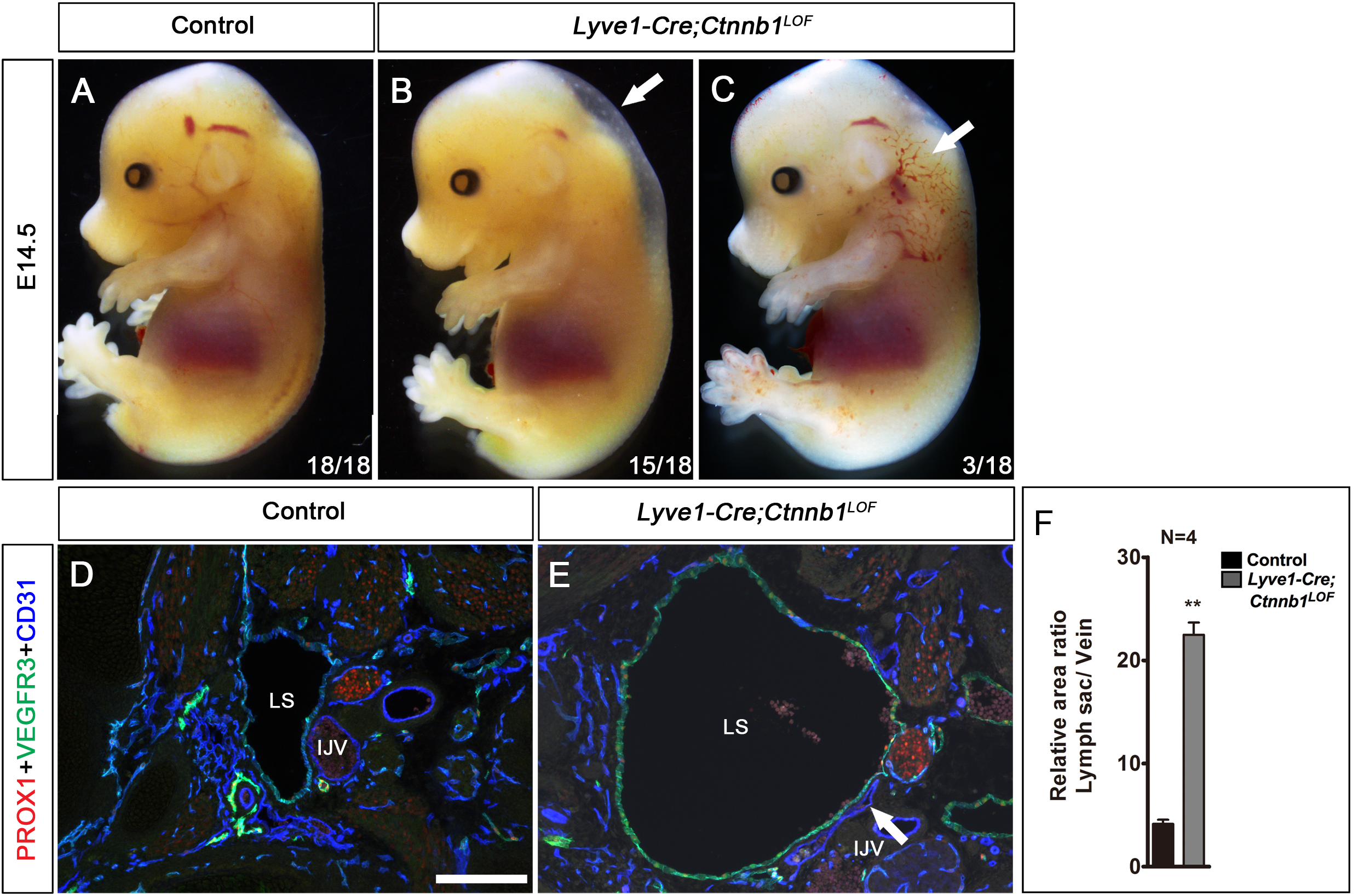

Supplement: Supplemental Material [file supp_gad.282400.116_Supp2.tif]

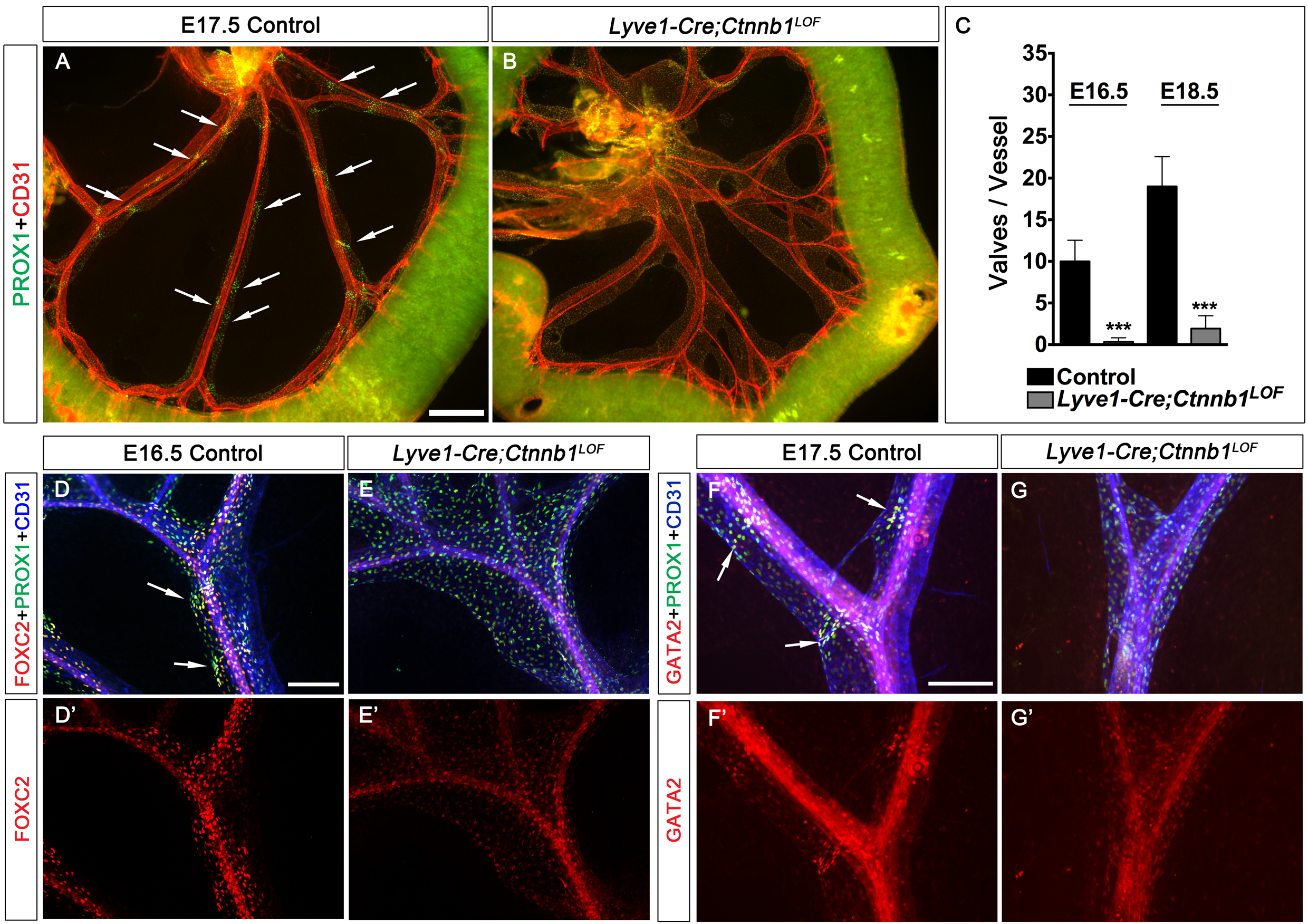

Supplement: Supplemental Material [file supp_gad.282400.116_Supp4.tif]

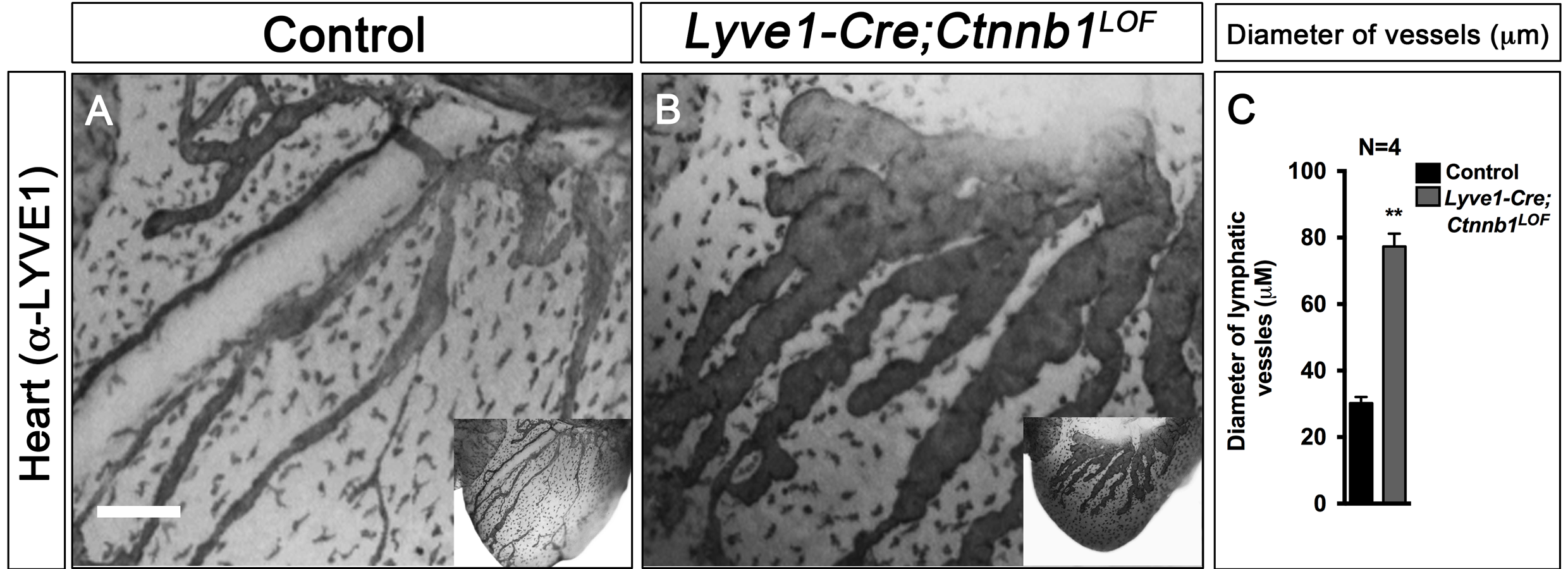

Supplement: Supplemental Material [file supp_gad.282400.116_Supp5.tif]

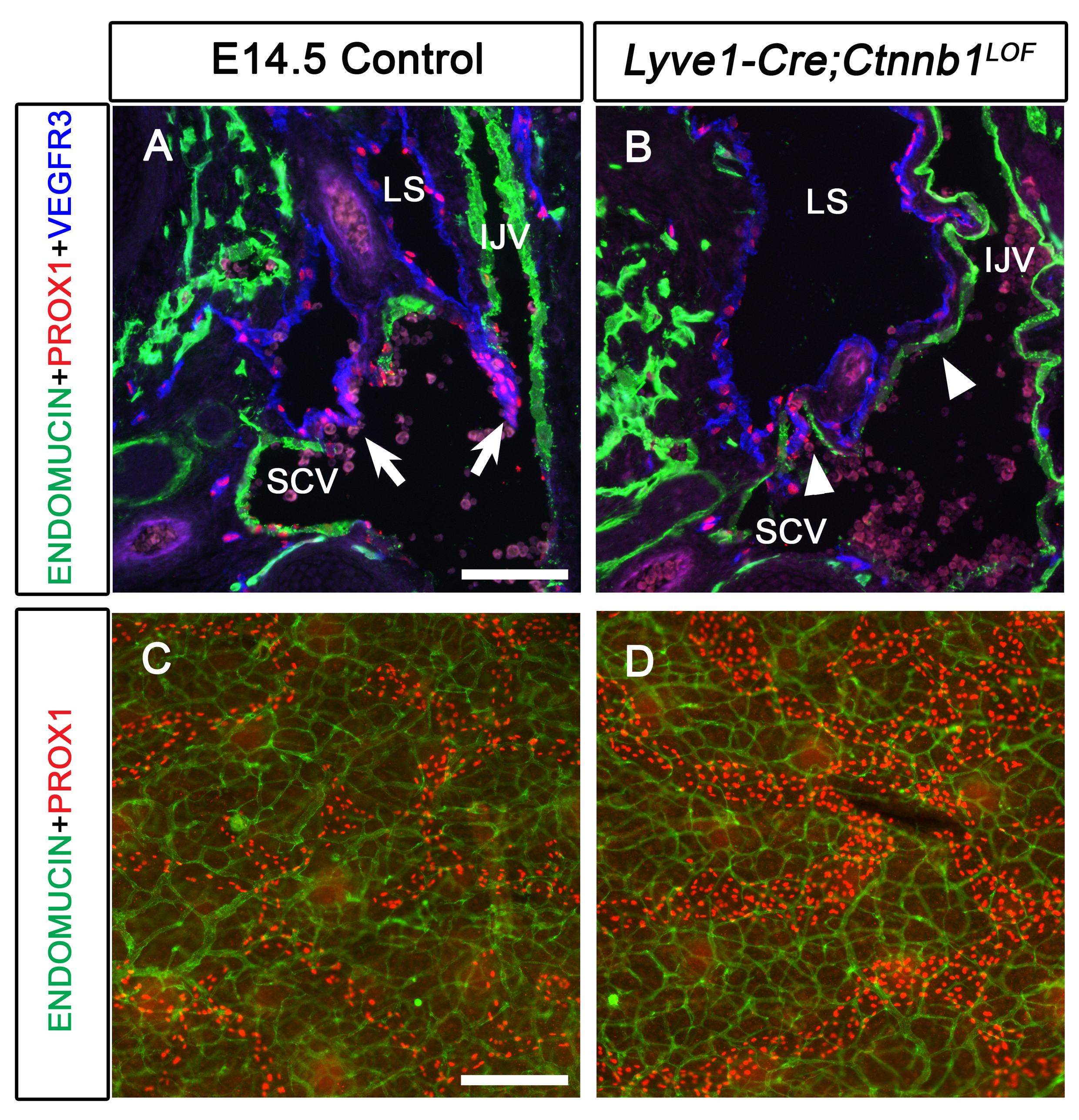

Supplement: Supplemental Material [file supp_gad.282400.116_Supp6.tif]

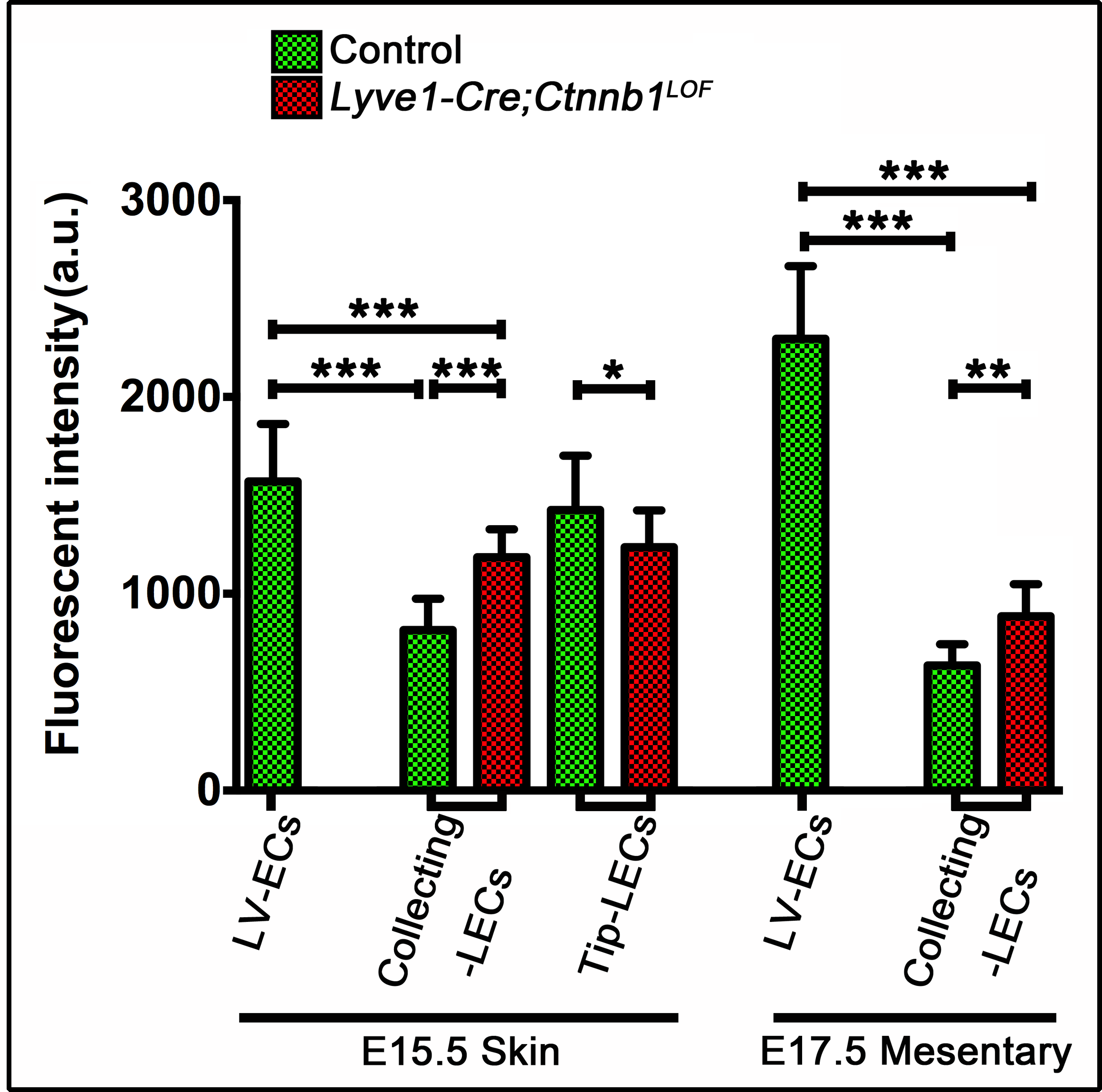

Supplement: Supplemental Material [file supp_gad.282400.116_Supp7.tif]

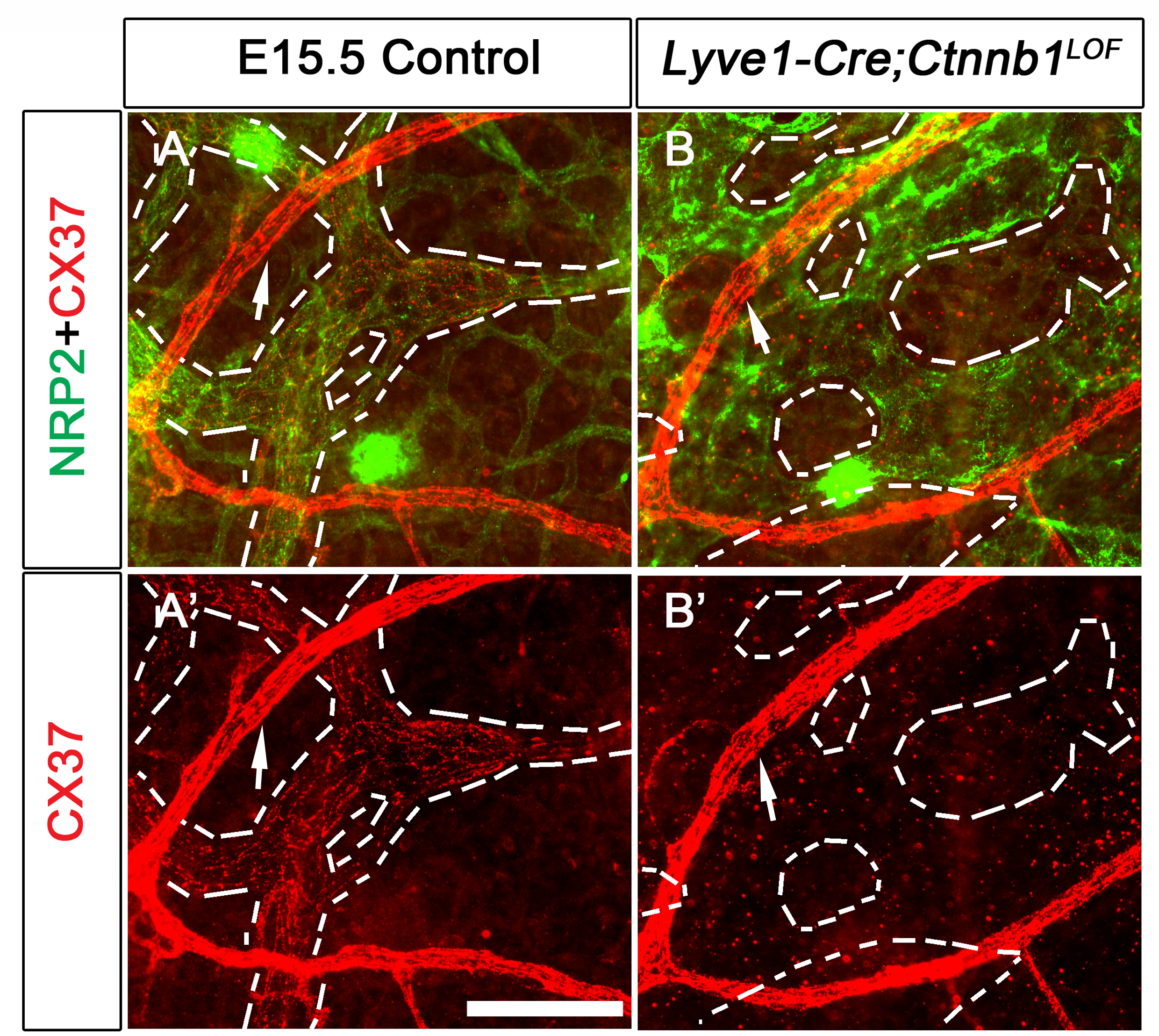

Supplement: Supplemental Material [file supp_gad.282400.116_Supp8.tif]

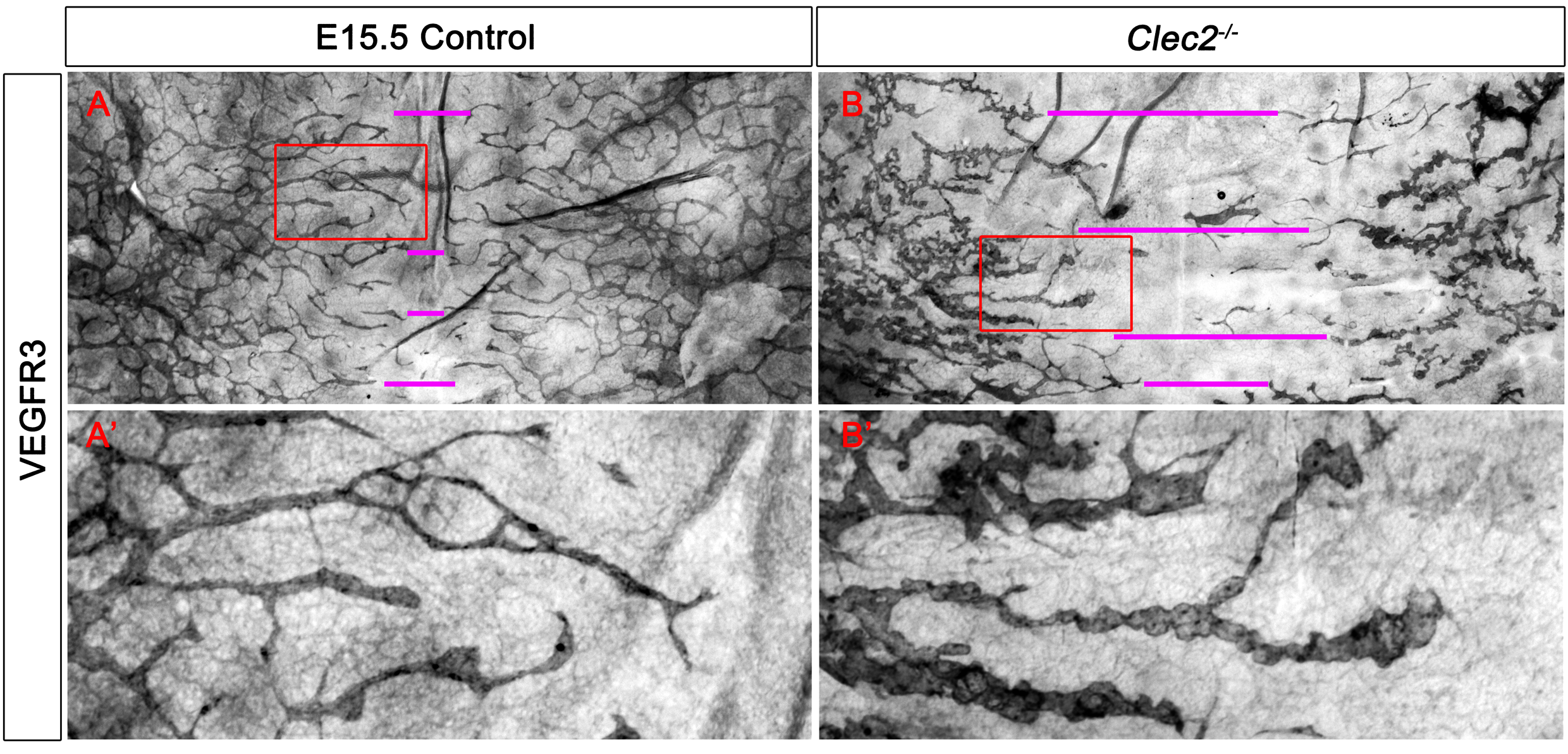

Supplement: Supplemental Material [file supp_gad.282400.116_Supp9.tif]

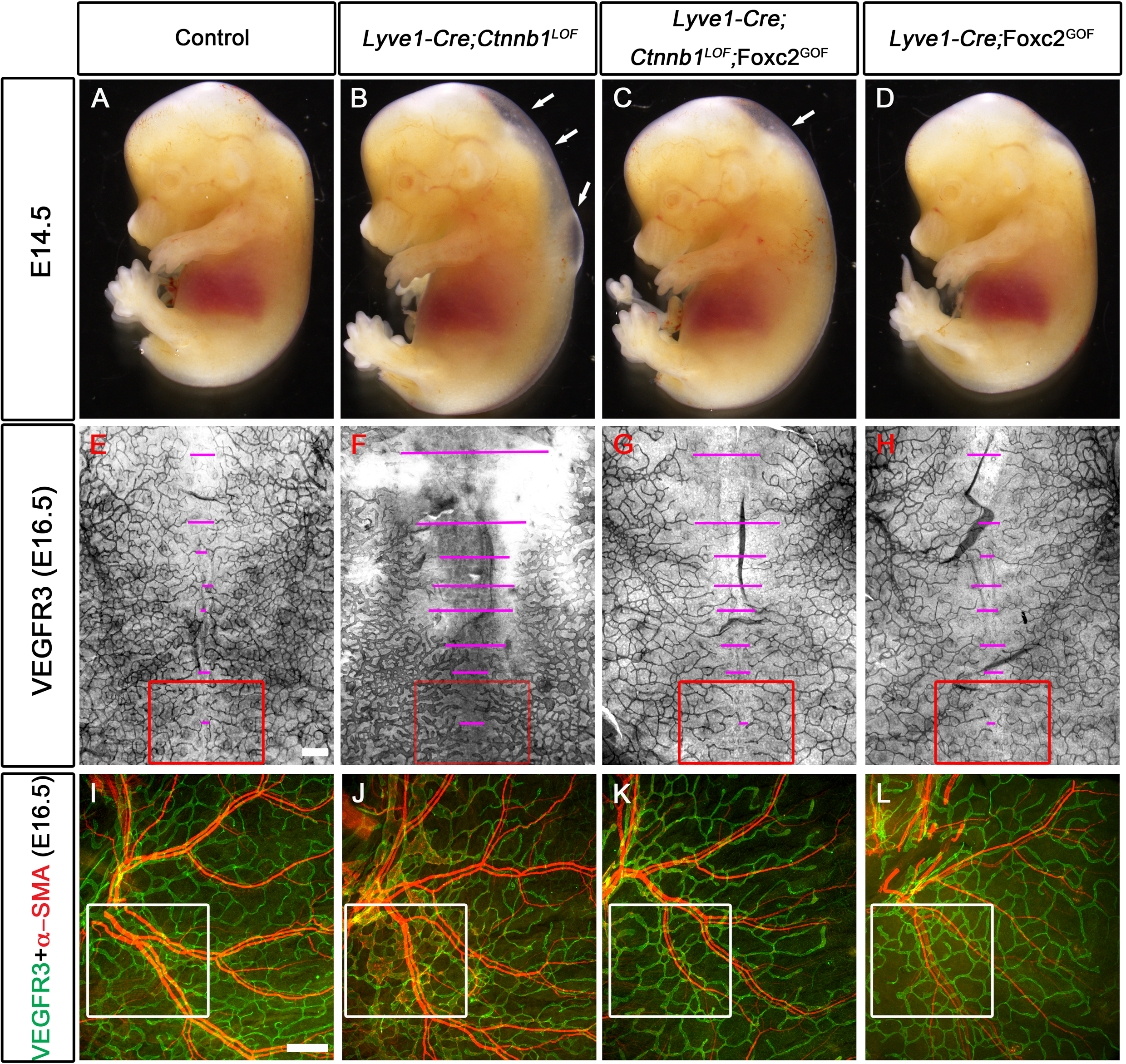

Supplement: Supplemental Material [file supp_gad.282400.116_Supp10.tif]

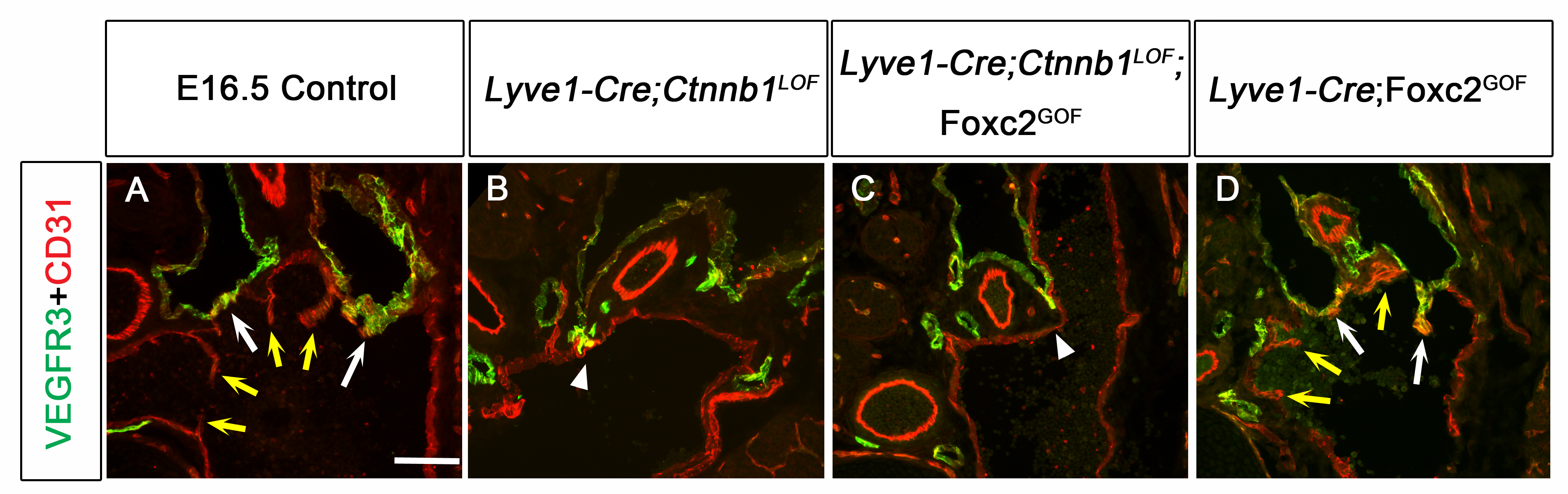

Supplement: Supplemental Material [file supp_gad.282400.116_Supp11.tif]

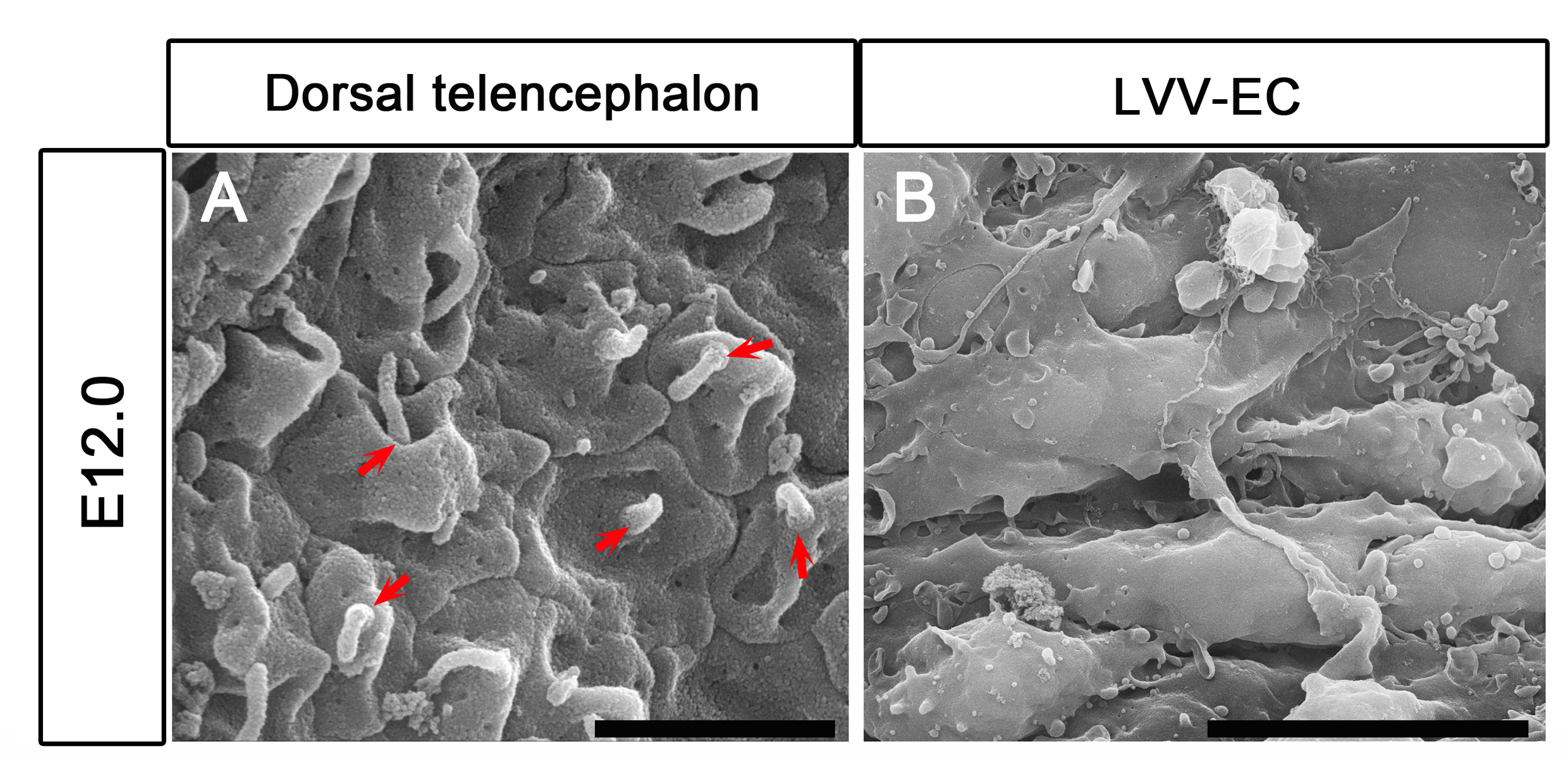

Supplement: Supplemental Material [file supp_gad.282400.116_Supp12.tif]
